# A symbiotic-like biologically-driven regenerating fabric

**DOI:** 10.1038/s41598-017-09105-4

**Published:** 2017-08-17

**Authors:** Neta Raab, Joe Davis, Rachel Spokoini-Stern, Moran Kopel, Ehud Banin, Ido Bachelet

**Affiliations:** 1 Bionics cluster, Augmanity, Rehovot, Israel; 2 The Mina and Everard Goodman Faculty of Life Sciences and the Institute of Nanotechnology & Advanced Materials, Bar-Ilan University, Ramat-Gan, Israel; 3 Department of Genetics and Wyss Institute for Biologically-inspired Engineering, Harvard Medical School, Boston, MA USA

## Abstract

Living organisms constantly maintain their structural and biochemical integrity by the critical means of response, healing, and regeneration. Inanimate objects, on the other hand, are axiomatically considered incapable of responding to damage and healing it, leading to the profound negative environmental impact of their continuous manufacturing and trashing. Objects with such biological properties would be a significant step towards sustainable technology. In this work we present a feasible strategy for driving regeneration in fabric by means of integration with a bacterial biofilm to obtain a symbiotic-like hybrid - the fabric provides structural framework to the biofilm and supports its growth, whereas the biofilm responds to mechanical tear by synthesizing a silk protein engineered to self-assemble upon secretion from the cells. We propose the term crossbiosis to describe this and other hybrid systems combining organism and object. Our strategy could be implemented in other systems and drive sensing of integrity and response by regeneration in other materials as well.

## Introduction

In contrast to living organisms, most everyday objects we use lack the ability to respond to damage and self-heal or regenerate. In cases where the cost of fixing an object is higher than the cost of replacing it, the latter option is usually preferred, enabled by a global industry that makes mass quantities of things such as electrical appliances, furniture, clothes, cars, and other similar mass-manufactured consumer products. This reliance may have very negative long-term environmental impact. It is therefore both interesting and important to hypothesize, whether objects can be made or programmed to respond and adapt like living organisms do.

As an example, fabric - prominent objects in human culture and technology - are flexible woven materials consisting of a network of natural or artificial fibres, made using diverse processes resulting in fabrics with a wide range of properties for various applications. The production of one kilogram of cotton for textile requires up to 20,000 litres of water^1^, and in addition consumes energy and chemicals (pesticides, fertilizers, etc.) leading to CO_2_ emission into the atmosphere and water pollution. It is estimated that over 11 million tons of textiles are trashed annually in the U.S alone^2^.

However, adaptive fabrics, or adaptive clothing, would self-heal in response to tear, and stretch or shrink as required; they could potentially also self-clean, remain protected from moisture, light, heat, and chemicals; and could potentially change their structure to allow physical protection (armor), evaporation, appearance, and more - all features exhibited by living organisms. Such fabrics could be the basis for a truly sustainable textile technology.

Self-healing and regeneration are highly complex properties based on two coupled phases: a sensory phase, in which a certain threshold of breach of structural integrity is detected by the system; and a synthetic phase, in which synthesis of new material is driven in response to the breach. While sensing per se may be easy to achieve (e.g. by integrating conducting fibers into textile such that tearing would result in a measureable change in the resistivity of a fabric segment), synthesis and its coupling to sensing is more challenging, especially if the purpose is achieving continuous, low-maintenance ability to self-heal, that does not require constant refilling and tuning of fabric monomer reservoirs.

Although self-healing and regeneration are critical components of sustainable textiles, reports on achieving them in artificial textile systems is extremely scarce. A recent study reported a polyelectrolyte layer-by-layer film coupled to squid ring proteins as a textile capable of suturing tears^3^. Several reports demonstrated fabrics capable of restoring their protective hydrophobic coating^4, 5^. A conducting fiber-containing, yarn-based supercapacitor has been shown in which magnetic attraction restores lost connectivity between electrodes^6^. While clearly exhibiting homeostatic behaviors, these reports lack other critical capabilities such as making new material de novo; most utilize artificial mechanisms deviating from biological strategies. In this work we designed and studied a preliminary draft towards true biological regeneration in fabric, based on integrating a bacterial biofilm into fabric, creating a structural hybrid between object and organism.

**Figure 1:**
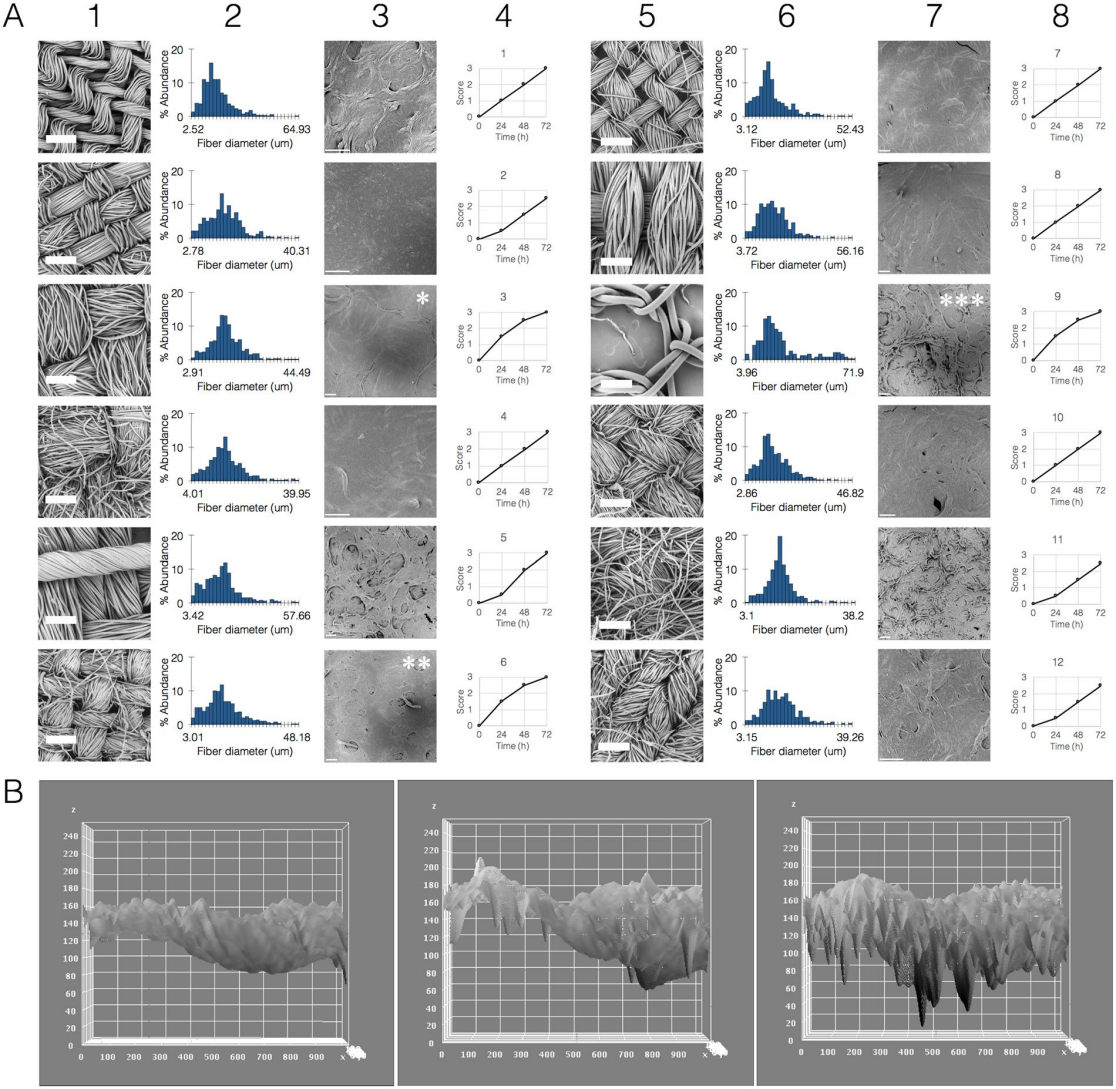
Fabric and hybrid analysis. (**A**) Fabric architectures (1st and 5th columns) were first examined without biofilms and unfixed using scanning electron microscope (SEM, thick scale bars = 300 *µ*m), and analyzed by FiberMetric (2nd and 6th columns showing fiber diameter distribution. Asterisks denote fabrics of various types which improved biofilm growth: *synthetic fabric made of polyester and cotton with large-sized threads and dense weave, **natural fabric made of cotton with small-sized threads and plain weave, ***synthetic fabric made of polyester with large fibres and loose weave. Fabric-biofilm hybrids were fixed after 3 days in culture and visualized in SEM (3rd and 7th columns, thin scale bars = 100 *µ*m). Biofilm viability was scored each day on a score of 0–3 (0 = no growth and clear medium, 3 = full growth and cloudy medium, 4th and 8th columns). Control biofilm without fabric was scored 0–1–2–3. Results representative of 3 independent repeats. (**B**) Image analysis showing simulated roughness of biofilm in each of the three representative fabrics (*^,^**^,^ ***, from left to right), demonstrating the correlation between weaving density and biofilm structure (x and y axes, location pixels; z axis, pixel intensity on a scale of 0–255).

Our first step was to study the feasibility of hybridizing the fabric with the biofilm such that the latter remains viable and metabolically active. For this work we chose *Bacillus subtilis* for being widely-studied, easy to work with and to genetically engineer, their biolfilms are characterized and can be reproducibly made. We cultured *B. subtilis* biofilms embedded inside pieces of fabric, screening a range of fabrics that included various materials (animal, plant, mineral, synthetic) and weaving patterns (fiber diameter, fiber density) (Fig. 1A, Supplementary note 1). All fabrics were compatible with biofilm growth and maintenance, with minor detected differences in viability or activity between groups **(**Fig. 1A **)**. Interestingly, some fabrics significantly improved the biofilm growth, while others inhibited it (Fig. 1A, panels marked with asterisks). Additionally, there was a clear correlation between fabric architecture and biofilm appearance; hybrids with less dense fabrics exhibited rough-surfaced, disordered biofilms, and ones within denser fabrics exhibiting the opposite phenotype (Fig. 1B, Supplementary note 1). This dependency suggests that the fabric serves as a structural framework or scaffold for the biofilm, and highlights the possibility of designing specific fabrics to achieve desired biofilm phenotypes and growth patterns.

In order to configure the response/synthesis role of the biofilm, the response of the biofilm to mechanical tear was mapped. While other responses have been previously reported^7–10^, the specific response to mechanical strain and tear, a likely natural scenario in bacterial evolution, has not. For this, total RNA was extracted from *B. subtilis* biofilms 5 min after subjecting them to mechanical tear, sequenced (Supplementary note 2) and analyzed to obtain the transcriptome response and identify tear-responsive elements. Rather than a single tear across a biofilm, and In order to maximize the signal, ~1,000 of small lateral tears were induced in the entire biofilm (average of ~3 tears per mm^2^ biofilm) using a custom-built array of metal needles positioned at high density and movable on the XYZ axes.

**Figure 2:**
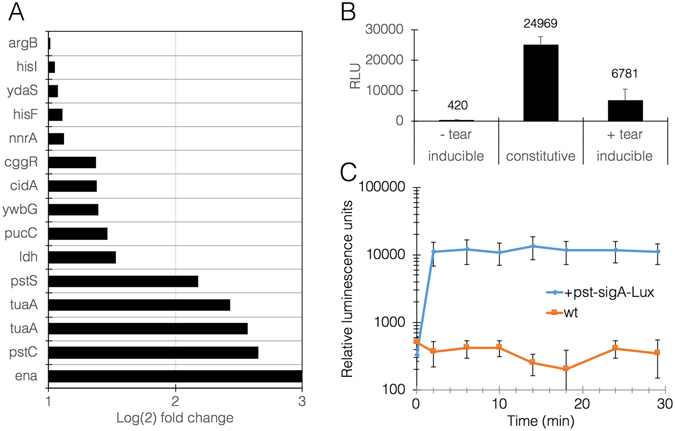
*B. subtilis* biofilm transcriptome response to mechanical tear and subsequent mounting of a reporter response. (**A**) graph representation of the most up- or down-regulated genes, in Log(2) of the fold change compared with untreated biofilm. Mean p-value for all changes is 0.007 ± 0.014. (**B**) A representative response of *B. subtilis* biofilms to tear, measured as relative luminescence units (RLU) derived from expression of the *lux*ABCDE operon under the control of *pst*-sigA promoter. This graph shows maximal signal achieved after 30 min. Data labels show mean values. *P*-value constitutive vs. +tear < 0.05; *P*-value -tear vs. +tear < 0.05. (**C**) A representative kinetic measurement of the response to tear driven by *pst*-sigA. Blue line represents *pst*-sigA reporter strain and orange line represents negative control (wildtype strain). *P*-value < 0.05 in all points (n = 3).

Transcriptome analysis highlighted specific pathways involved in the response to tearing, particularly cell wall remodeling (teichuronic acid and peptidoglycan biosynthesis) and cell division (phosphate uptake, nucleotide and aminoacyl-tRNA biosynthesis) (Fig. 2A, Supplementary note 3 & 4). Tearing also induced activation of the sigma M regulon, which has been shown to operate in response to cell wall stress induced by antibiotics and other chemical stimuli^11^. Interestingly, population control genes such as *skf* (sporulation killing factor) and *sdpA/B* (sporulation delaying proteins) were inhibited, suggesting a potential disinhibition for purposes of populations regrowth. These patterns were highly reproducible in independent experiments. Based on these findings, 5 promoters were identified and selected as candidate tearing-induced drivers of fabric synthesis (Supplementary note 5). All 5 showed at least 8-fold increase in expression upon stimulus while maintaining minimal expression unstimulated. Test drivers were constructed in which each of the 5 promoters was placed to control expression of the *lux*ABCDE operon. Biofilms transformed with a selected promoter, pst_sigA, responded well to tearing **(**Fig. 2B,C **)**.

Next, we turned to designing the synthetic part of the system. The choice of genes for fabric synthesis was guided by mechanistic simplicity: a single gene, and the ability to self-assemble into a functional fiber under specific conditions. Arthropod silks have been known for millennia and are still considered industrial benchmarks today^12^. However, silks from spider species or from the silkworm *Bombyx mori* require complex weaving organs, making them unsuitable for the purposes of the present design^13, 14^. For this reason, silk from other sources was examined. Raspy crickets (*Gryllacrididae*) produce silk for building leaf shelters^15^. Recently, several genes encoding cricket silk were cloned from the cricket labial glands, and their partial sequences include alanine/glycine/serine-rich repeats typical of silk proteins from other species^16^.

**Figure 3:**
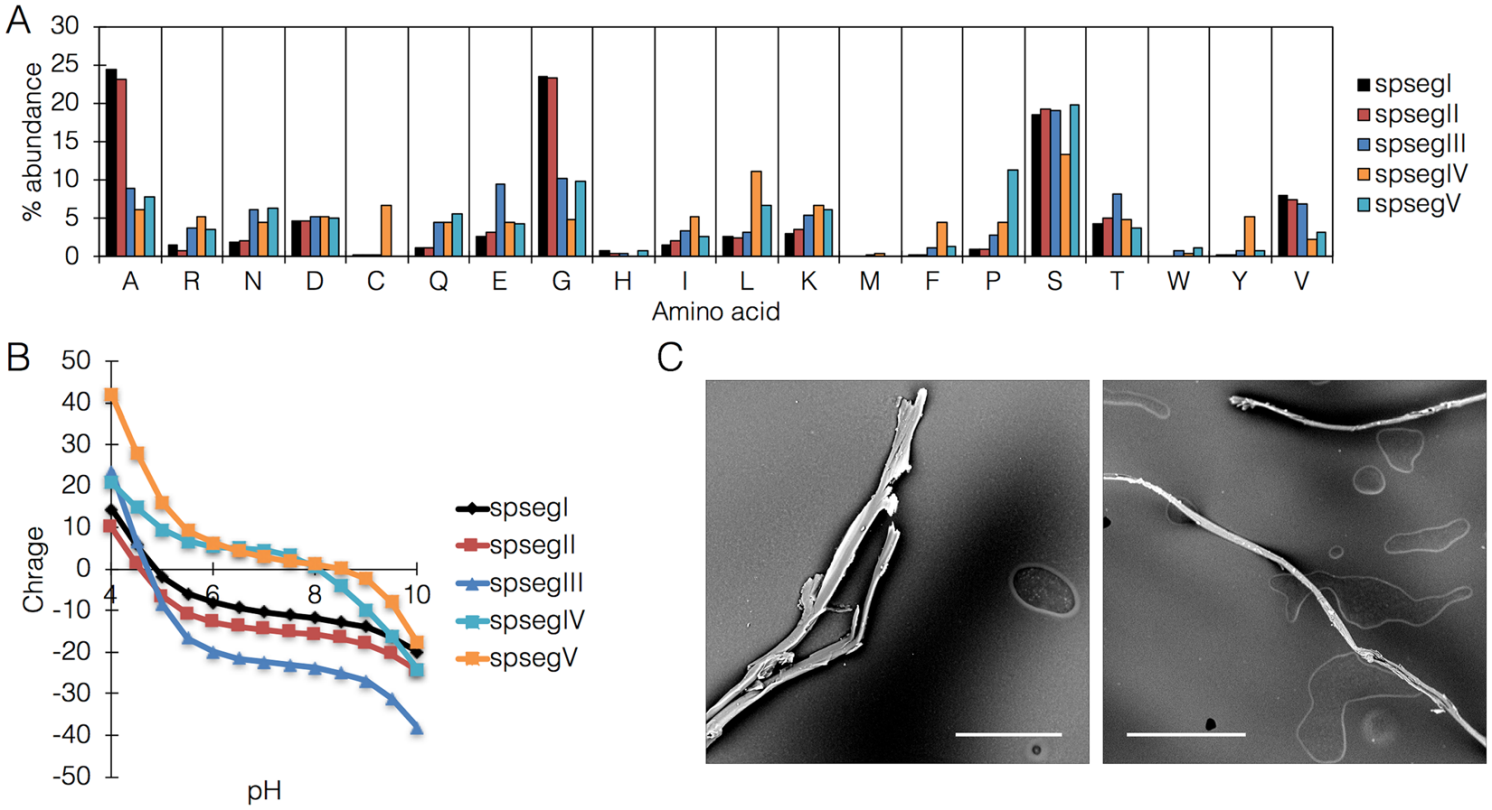
Silk protein analysis and self-assembly. (**A**) amino acid distribution in all 5 silk protein segments. (**B**) calculated net charge on protein surface. (**C**) SEM images showing assembled protein fibers following concentration by dehydration and acidification to approximately the calculated isoelectric point. Size bars = left, 50 *μ*m; right, 100 *μ*m.

In order to evaluate the suitability of these proteins for the synthetic module, segments of these protein sequences (termed *spseg*I/II/III/IV/V) were selected and fused to histidine tags for expression vector construction (Supplementary note 6). Isoelectric points of the protein segments were calculated, with an interesting distribution into two groups, an ‘acidic’ group containing 3 proteins with pI at ~5.0, and an ‘alkaline’ group containing the remaining 2 proteins with pI at ~8.0 (Fig. 3A). The proteins were expressed in both insect and bacterial systems (Supplementary note 7), in both cases showing efficient assembly upon cumulative acidification and dehydration (increasing protein concentration) (Fig. 3B) into fibers of a mean diameter of 10 um, with an elastic modulus of 4.54 GPa and tensile strength of 617 MPa.

**Figure 4:**
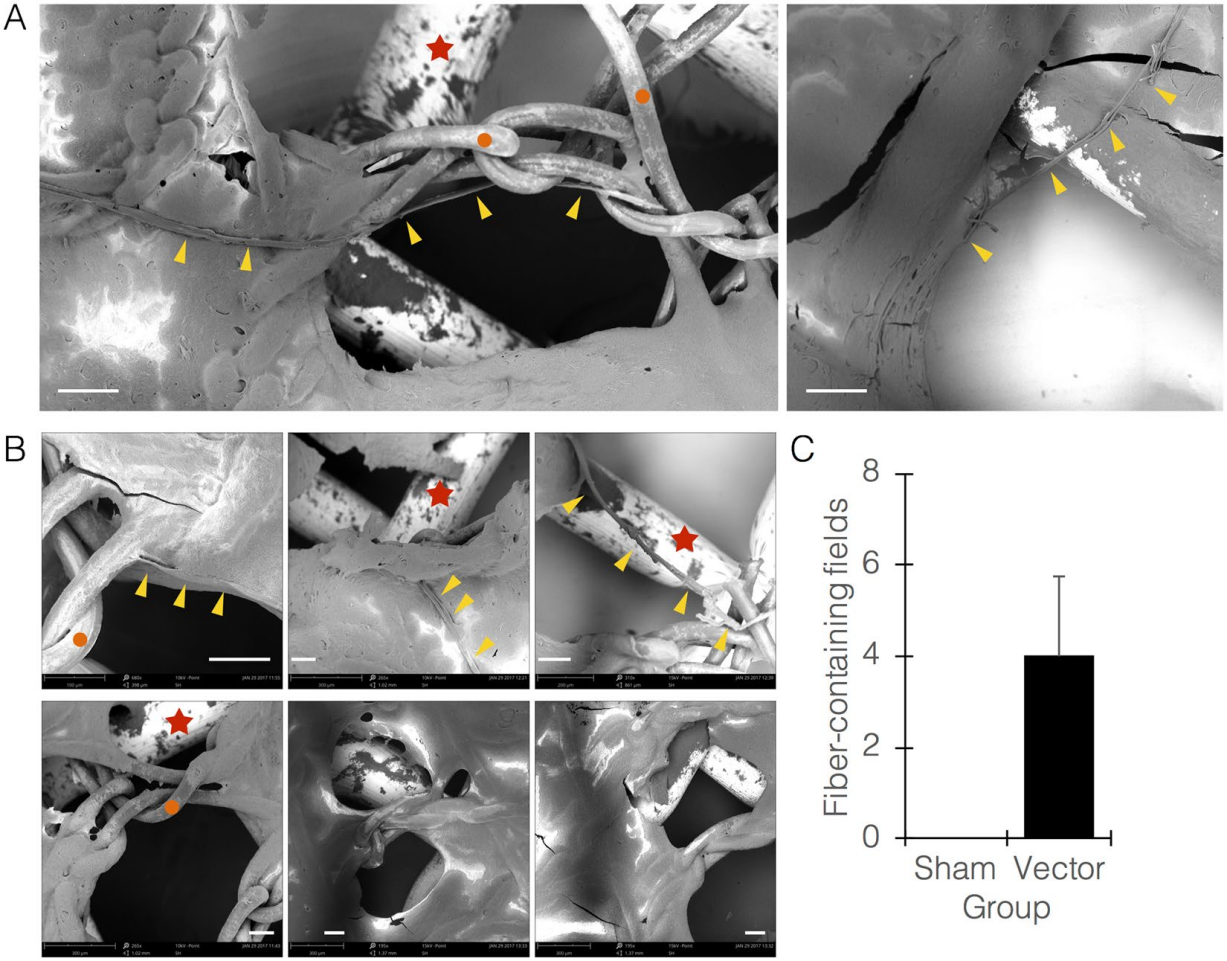
Regenerating fabric system. (**A**) Representative SEM images of regenerating fabric at t = ~15 minutes following tear of fabric-biofilm hybrid. Yellow arrowheads point at newly-made and assembled silk fibers. Red stars point at metal net discs used for imaging. Orange circles point at fabric fibers. (**B**) images of torn regenerating hybrids (top 3 panels) vs. torn hybrids made with wildtype biofilms (bottom 3 panels), the latter showing no newly-made fibers. All images were taken at tear region. (**C**) Quantitation of imaging fields visualized by light microscopy (sham, wildtype biofilms; vector, engineered biofilms). All size bars = 100 *μ*m.

Finally, a selected protein segment was placed under the control of a selected promoter, and the fabric-biofilm hybrids were prepared using *B. subtilis* transformed with the constructed vector. Five min following tearing of the hybrid without submerging or lifting it from the well, and under acetic acid-mediated slight acidification of the medium (pH ~6.0), we observed assembled single fibers, originating within ±20 um from the rim of the tear region (Fig. 4A; Supplementary movie S12). It is noteworthy that the time periods we used are short for a secreted protein to accumulate in significant amounts outside the cell, however the observed rapid assembly is caused by cell lysis due to impact with the acid. Interestingly, silk protein assembly occurred mostly along and around fabric fibers, suggesting that the assembly process is more efficient on the fiber surface, hence the fabric serves as scaffold or guide for the process. Hybrids made with wildtype biofilms were torn as well, without any apparent response (Fig. 4B,C). The assembled silk fibers showed a strong (~30%) nitrogen band, while the scaffold fabrics showed only carbon and oxygen bands (~70% and 30%, respectively).

The aim of the present study was to demonstrate plausibility, rather than yield an optimal design. Much work is still needed, mainly in optimizing the expression and assembly efficiency in order to achieve full coverage of the torn region, the fabric material and structure, and the fabric-biofilm interface. It is hypothesized that specific fabrics could be designed, woven, and tested for desired outcomes. Examples include having the fabric retaining liquid medium for an extended period, or the fabric itself being coated by a layer of food source such as starch, which the bacteria can metabolize. An effect of fabric type on biofilm growth was observed in this study, with potential reasons being retention or concentration of nutrients in the fibers. In addition, our observations suggest that the fabric serves as a structural scaffold to both the biofilm and protein fiber assembly, a function that can also be encoded in the architecture of designed fabrics.

The draft concept we describe highlights interesting questions and potential solutions on the technical and conceptual levels. For example, the compliance of individuals to wear or use a bacterially-loaded fabric could be improved by engineering a better interface in which the bacteria are contained within the fabric fibers and do not form a separate layer. Another example is that the biofilm is not expected to survive laundry. A potential solution to this issue could be to add dried bacteria or spores as a post-laundry or pre-wearing reagent, effectively reloading the fabric with regeneration capacity. Particularly, current progress in synthetic biology encourages expanding the present concept even further: the bacteria within the fabric could be engineered to produce scent, pigments, or even antibiotics against skin-infecting bacteria.

Lastly, the coupling of genes encoding sensing of environmental cues with genes encoding material synthesis highlights a novel category of machines, robots, and useful tools. From adhesives and metals to fractal materials and optic fibers, biological organisms fabricate an astonishing variety of materials currently available to us only by complex engineering processes, and do so in their natural environment under relatively ambient conditions. These possibilities are discussed in Supplementary note 8.

This work strengthens the possibility of a novel class of hybrid systems - objects with fully integrated biological components. Recently, a similar system was reported that employes hygroscopic properties of bacteria to design a ventilating fabric^17^. We propose the term crossbiosis to describe these systems (after the term symbiosis), to stress the fact that a living organism and a nonliving object are combined to create a new entity with synergistic properties.

## Methods summary

### Bacterial strains and growth conditions

*B. subtilis* (strain NCIB3610) harboring a chromosomally encoded *gfp* reporter gene and Chloramphenicol (CM) resistance as well as the Wild Type (WT) strains were a kind gift from Ilana Kolodkin-Gal (Weizmann Institute of Science). For biofilm formation, bacteria were cultured in Minimal medium (MSgg) as previously described at 23 °C for 72 hours^18^.

### Fabrics

Several types of Fabrics (silk, 100% cotton, synthetics, mixes, Gütermann threads and others) were purchased from Gudes and The Sewing Center LTD. (Israel). Fabrics were cleaned by washing in filtered deionized water and sterilized by soaking in 70% ethanol followed by in-plate UV irradiation. Fabrics were stored at room temperature and humidity in closed sterilized culture plates.

### Microscopy and image analysis

Structural analysis of fabric and biofilm architecture and bacterial growth and integration into the fabric were measured using PhenomWorld ProX SEM with EDS module. Samples were prepared with minimum manipulations. Fresh and unfixed samples were dried in a low vacuum desiccator for ~20 minutes, until biofilms were dry but not cracked.

Images were analyzed using ImageJ (Fiji) software. For analysis all images used were formatted to 8-bit binary (grayscale). SEM information bars were cropped out of the figures before analysis. Histograms and surface plots were done using default parameters and all actions were applied on all figures identically.

### Transcriptome response to tear

Biofilms were subjected to mechanical tear by a custom-built moving array of sterilized, round-tip stainless steel needles (200 micron tip diameter, 1 mm tip-to-tip distance) introduced into the biofilm to a depth of 4 mm to ensure simultaneous uniform tearing in as many points as possible. RNA was extracted from biofilms 5 minutes following tearing using FastRNA PRO™ BLUE kit (MP Biomedicals) and compared between untreated and treated groups. Experiments were done in triplicates and quality and quantity of RNA was evaluated using spectrophotometry on a Nanodrop 2000 instrument and bioanalyzer (Agilent 2100). Library preparation (TruSeq RNA without the oligo-dT stage) and sequencing (SR 60 v4 High Output) were performed at the Nancy and Stephen Grand Israel National Center for Personalized Medicine, Weizmann Institute of Science, Israel.

### Bioinformatic analysis

Quality control on RNA seq reads was done using FastQC^19^. Adapters were removed using Cutadapt^20^, discarding reads with less than 40 bases after adapter trimming. Reads with more than 50% polyA/T were removed using a custom-written script. Counting was done using HTSeq^21^ and gene annotation was based on Ensembles B. subtilis GTF. Differential expression analysis was done using DESeq. 2^1, 3, 6^, with no independent filtering and beta prior. Raw p-values were adjusted for multiple testing using FDR(BH).

### Plasmid and strain construction

pBS3C*lux*-[RFP] integration plasmid for gram positive bacteria^22^ was a kind gift from Daniel R. Zeigler (The Bacillus Genetic Stock Center). RFP gene was replaced with *pst-sigA* promoter. All primers used in this study are listed in Supplementary note 9 in the supplemental material. Genome integration transformations of *B. subtilis* into *sacA* locus were carried out using the competent strain DK1042^23^, an identical strain to NCIB3610 except for a single point mutation that inactivates *comI*, a naturally-occurring plasmid-borne competence-suppressing gene, and increases competence 100-fold. Competent cultures were grown in diluted modified competence (MC) medium as previously described^23^ and plated on LB plates containing 5ug/ml chloramphenicol to select for transformants. Integration of plasmids into *sacA* locus of the *B. subtilis* genome was checked with colony PCR (Supplementary note 9).

### Luciferase assay

Luciferase activity of strain harboring chromosomally encoded P_*pst*-sigA_-*lux*ABCDE was assayed using a SynergyHTX multi-mode reader from BioTek® (Winooski, VT, USA). The reader was controlled using the software Gen5. Culture volumes were 100 μl per well in a bioluminescence-compatible 96-well plate, and incubation occurred at 23 °C for 72 hours. In all wells biofilms were formed. Positive control strain harbouring a constitutive strong promoter P_veg_^22^ was used to adjust sensitivity for optimum results. Plate was monitored for luminescence prior to mechanical tear. Once biofilms were subjected to tear (as described above), luminesce was monitored for 30 minutes in 4-minute intervals. Experiment was done in triplicates.

### Gene design and synthesis

Glycine/Serine/Alanine-rich segments (termed spseg*I* to spseg*V*) from silk genes sequenced from Australian raspy crickets^16^ were selected for their chemical properties and assembly potential^24, 25^. For initial expression, protein segments were synthesized de novo using *E. coli* codon optimization and cloned into a Clontech pBE-S vector (a system optimized for *Bacillus* secreted proteins). For expression in arthropod cells (S2 drosophila cells), genes were recoded for eukaryotic expression and recloned into the pMT/BiP/V5-HisA vector, containing the N-terminal signal sequence from the insect BiP gene, a C-terminal V5 epitope, and a C-terminal 6His tag for purification. All plasmid maps can be found in Supplementary note 10.

### Flow cytometry

S2 cell count and viability assays were done on a BD Accuri™ C6 flow cytometer. Cells were checked routinely every 3 days, before subculturing and transfections. For analysis, S2 cultures were diluted 1:10 into PBS and viewed in forward scatter/side scatter channels. For viability analysis, propidium iodide (PI) was added to a final concentration of 0.1 ng/*µ*L and cells were vortexed briefly. Silk gene transfection success and protein production were evaluated by intracellular flow cytometry as follow: cells were fixed with 2% formaldehyde, then perforated by a brief incubation in frozen 100% methanol. Cells were washed with FX buffer (0.1% w/v bovine serum albumin, 0.05% w/v sodium azide in PBS, pH 7.4), and incubated with primary (anti-V5 epitope) and secondary antibodies with washes in between.

### Protein expression and purification

Silk segments were first expressed in *E. coli* to assess assembly into fibers, and extracted using a commercial kit. Fibers were analyzed visually and by SEM. S2 cells were used for large scale purification, and sIlk segments were purified on a Ni-NTA column in an AKTA-Start instrument and evaluated by SDS-PAGE and western blot. Western blotting was performed on a Bio-Rad blotting system using commercially available reagents and standard protocols; membranes were developed using Novex HRP Chromo kit. Purified protein was dialyzed overnight into PBS on 12,500 Da molecular weight cutoff dialysis tubes, concentrated with 3 sequential runs on Amicon 0.5 mL 10,000 Da cutoff tubes, and acidified with 17 M acetic acid to a pH of ~5.8.

### Tearing experiments

Fabric-biofilm hybrids on the medium-air interface were torn as described above, and were let to sit for 5–15 minutes before acidification of the medium by adding concentrated acetic acid and rapid mixing to achieve a pH of approximately 6.0. Microscopy (light and SEM) was then used to count occurrences of assembled silk fibers, and EDS was used to analyse elemental composition and discriminate between these fibers and the scaffold fabrics.

## Electronic supplementary material

Supplementary information
