# Supplementary material for: A symbiotic-like biologically-driven regenerating fabric
Source: Sci Rep. 2017 Aug 17;7:8528. doi: 10.1038/s41598-017-09105-4 (PMC5561142; doi:10.1038/s41598-017-09105-4)
Supplement: Supplementary file 1 — Supplementary information [file 41598_2017_9105_MOESM1_ESM.pdf]

A symbiotic-like biologically-driven regenerating fabric

Neta Raab[1,2], Joe Davis[3], Rachel Spokoini-Stern[1,2], Moran Kopel[1], Ehud Banin[2], Ido Bachelet[1,4]

1. Bionics cluster, Augmanity, Rehovot, Israel.
2. The Mina and Everard Goodman Faculty of Life Sciences and the Institute of Nanotechnology & Advanced Materials, Bar-Ilan University, Ramat-Gan, Israel.
3. Department of Genetics and Wyss Institute for Biologically-inspired Engineering, Harvard Medical School, Boston MA, USA
4. Address for correspondence: 8 Hamada St., Rehovot 7670308, Israel. Tel +972-546216664. E-mail: dogbach@gmail.com

Supplementary notes

Supplementary note 1: Fabric and hybrid analysis
Supplementary note 2: Transcriptome table
Supplementary note 3: Bioinformatics/QC of RNAseq
Supplementary note 4: Pathway enrichment
Supplementary note 5: Promoter sequences
Supplementary note 6: Silk protein sequences
Supplementary note 7: Protein expression in insect and bacterial systems
Supplementary note 8: Regenerating fabric system
Supplementary note 9: on hypothetic crossbiotic systems
Supplementary note 10: Primer table
Supplementary note 11: Plasmid maps
Supplementary note 12: Culture media and protocols

List of supplementary figures and tables

Fig S1: fabric architectures
Table S1: fabric-biofilm characterisation
Fig S2: QC of RNA seq reads
Fig S3: HC counts
Fig S4: Principal component analysis
Fig S5: differential gene expression
Table S2: pre-processing of reads
Table S3: mapping summary
Table S4: transcriptome table
Fig S6: DAVID analysis
Table S5: KEGG ortholog list for iPATH
Table S6: Tear-responsive elements
Table S7: crossbiotic systems
Fig S7: Assembly of silk fibers from transformed *E. coli*
Fig S8: Silk fiber structures of single segments and a combination product
Fig S9: S2 cell culture and transformation
Fig S10: Protein purification and analysis
Fig S11: Silk protein expressed in S2
Movie S12: Regenerating fabric system, SEM imaging
Fig S13: cost range of usable materials
Table S8: primers used in this study
Table S9A,B,C: culture medium ingredients
Table S10: 10X MC ingredients
Fig S14: plasmid map, pBE-S-SPsegII
Fig S15: plasmid map, pMT-BiP-V5-HisA-SPsegII
Fig S16: plasmid map, pBS3Clux-pst_sigA
Fig S17: plasmid map, pINsilkII

Supplementary note 1: Fabric and hybrid analysis

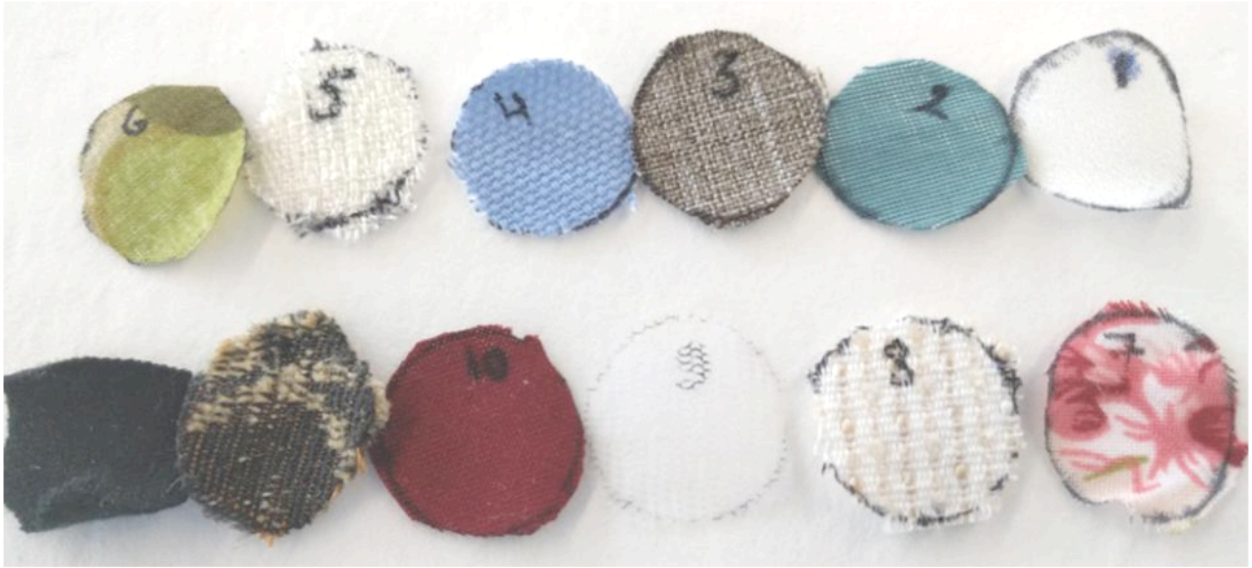

Figure S1. Partial selection of fabrics represented in this study.

1 - 70% chiffon 30% polyester
2 - 100% nylon
3 - 90% polyester 10% cotton
4 - 55% acrylic 45% nylon
5 - 55% cotton 45% polyester
6 - 100% cotton
7 - 90% nylon 10% cotton
8 - canvas
9 - 100% polyester
10 - 55% linen and 45% cotton
11 - 100% cashmere wool
12 - 85% spandex 15% polyester

Table S1. Fabric-biofilm characterization

	Fabric 6 (**)	Fabric 3 (*)	Fabric 9 (***)
Fabric material	Cotton	Cotton and polyester	Polyester
Thread size (μm)	15±12	15±10	18±12
Density (white:black px ratio)	1.6	1.1	0.4
Biofilm roughness	+	+	+++

*density count using Fiji => binary black background; adjust threshold; selection (white); measure; selection inverse (black); measure.

Supplementary note 2: Bioinformatics/QC of RNAseq

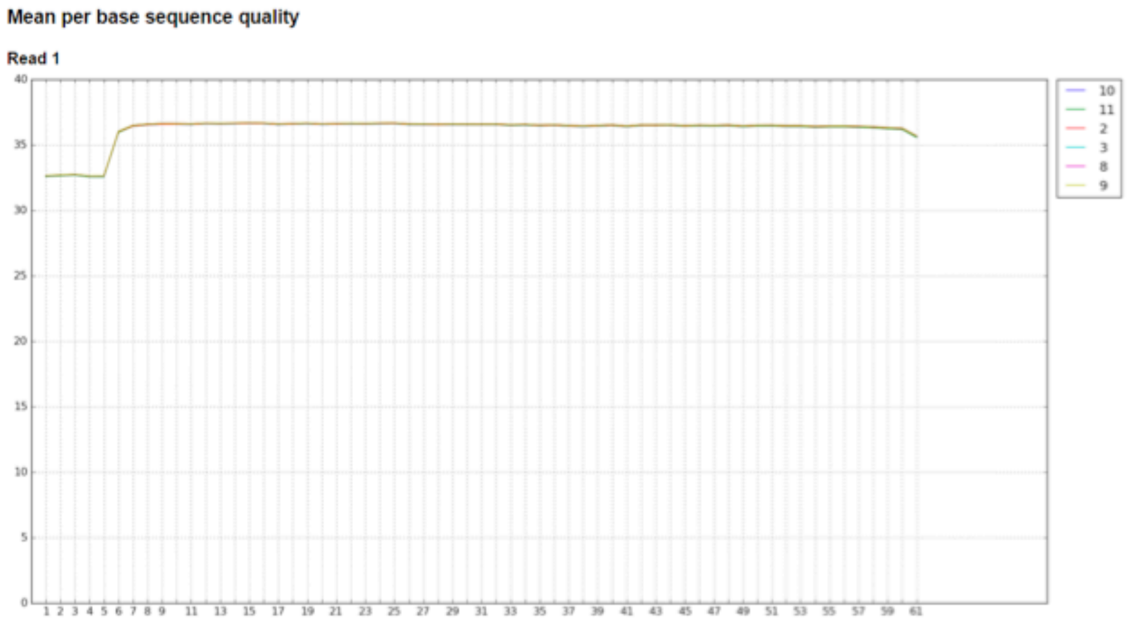

Figure S2. Quality control of RNA seq reads, performed using FastQC. Graph shows counts of reads length for all samples. Base quality is good. [ ]

Table S2. Pre-processing of reads

Sample	Condition	Reads	% Trimmed	% Poly A/T	# Reads after pre-processing
2	WT	45,835,100	0.20%	0.08%	45,737,166
3	WT	54,865,647	0.20%	0.08%	54,804,930
8	WT	50,848,481	0.20%	0.01%	50,794,691
9	T	46,945,025	0.10%	0.01%	46,149,689
10	T	48,977,290	0.10%	0.01%	48,842,321
11	T	51,358,845	0.10%	0.05%	51,240,417

Adapters were removed using cutadapt. Reads that had a length less than 40 bases after the adapter trimming were discarded, as well as reads with more than 50% polyA/T.

Table S3. Mapping summary

Sample	Condition	% Uniquely mapped reads
2	WT	98.40%
3	WT	98.40%
8	WT	98.50%
9	T	98.50%

10	T	98.20%
11	T	98.30%

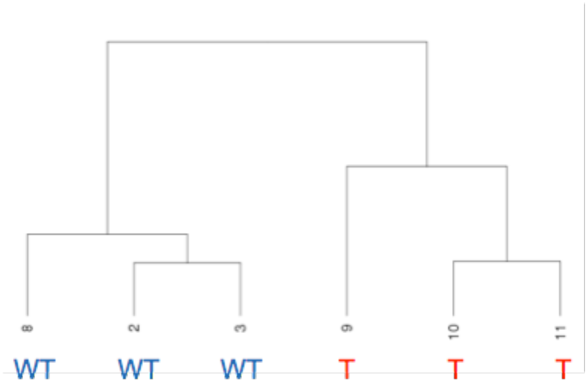

Figure S3. HC counts. Counting was done using HTSeq and gene annotation was based on Ensembles *B. Subtilis* gtf: (ftp://ftp.ensemblgenomes.org/pub/bacteria/release-30/gtf/bacteria_0_collection/bacillus_subtilis_subsp_subtilis_str_168/Bacillus_subtilis_subsp_subtilis_str_168.GCA_000009045.1). HC was done on 100 most varying genes (T – samples that underwent tearing, WT – control samples).

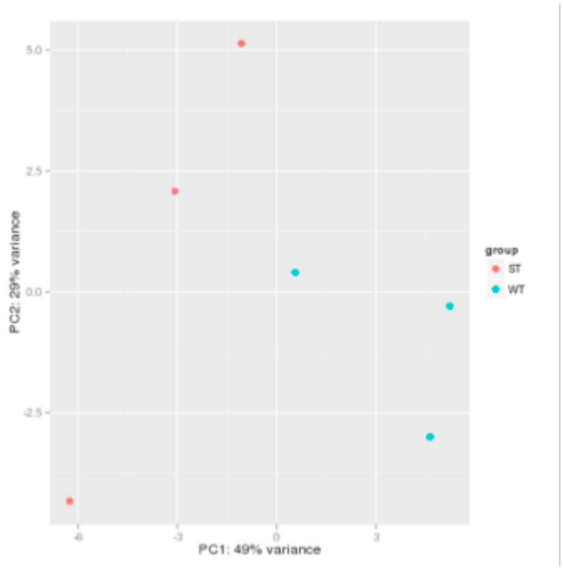

Figure S4. The statistical method, principal component analysis, was used to evaluate the variance between all samples. Clusters of triplicates - control group and experiment, were found.

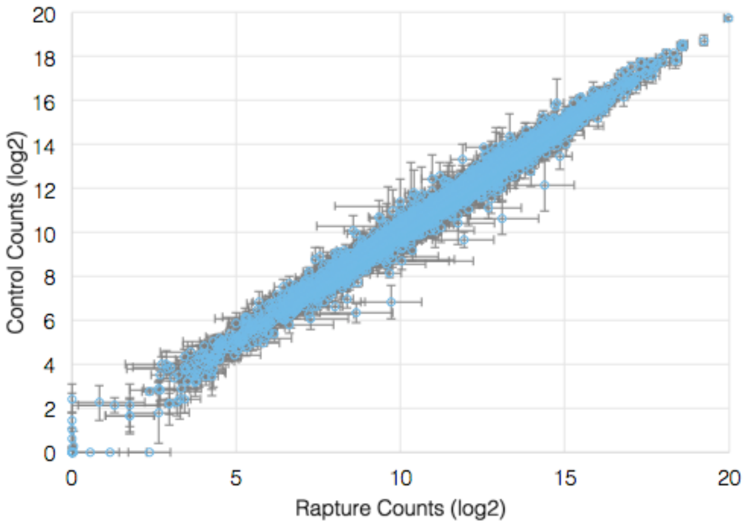

Figure S5. Differential gene expression of torn vs. control biofilms based on RNA seq.

Supplementary note 3: Transcriptome table

The following table lists raw data values of gene expression for torn vs. control biofilm. Numbers under “tear” and “control” columns are sample IDs.

Table S4. Transcriptome table

		Tear			Control						
Gene_ID	Gene_Name	9	10	11	2	3	8	baseMean	log2FoldChange	pvalue	padj
BSU12000	manR	1356	1502	1274	3166	3751	3466	2378.21252326146	1.22775631765743	2.47E-32	1.06E-28
BSU35690	ggaA	24598	28904	24723	57884	61524	49518	40680.8536088913	1.01591481989121	6.63E-14	9.48E-11
BSU40610	trnY-Asp	360	493	321	976	1119	920	686.072851659643	1.26591026119556	7.62E-13	5.45E-10
BSU03050	ldh	965	643	772	287	351	255	556.326022069365	-1.52649980440284	1.78E-12	1.06E-09
BSU35698	yvzI	1162	1609	1114	3288	3853	2786	2261.98043465887	1.25882201073143	1.97E-12	1.06E-09
BSU18860	yozH	248	274	228	572	571	506	395.275347830953	1.04190796400333	4.48E-11	1.92E-08
BSU40600	trnY-Asp	538	850	581	1667	1842	1415	1129.84301186903	1.23237810306344	1.37E-10	5.35E-08
BSU38320	cidA	216	295	238	118	96	92	178.281682081823	-1.37723419152902	1.94E-10	6.40E-08
BSU20450	yorA	938	1046	946	2634	2566	1798	1636.15446309779	1.16482059784445	8.63E-10	2.32E-07
BSU20430	yorC	150	222	178	586	586	383	345.628799418969	1.41217526924639	1.57E-09	3.97E-07
BSU38330	ywbG	295	387	282	159	116	114	228.923100113098	-1.38982256650076	2.73E-09	5.86E-07
BSU21460	bdbA	285	388	447	1371	1644	667	784.85724712148	1.62099023650937	1.71E-07	2.15E-05
BSU21360	yomH	158	185	154	504	616	287	311.633543397729	1.40239850907806	2.60E-07	3.01E-05
BSU27630	yrvD	2356	2570	2418	6458	6378	3907	3972.98279623634	1.09750440450693	3.64E-07	3.78E-05
BSU40160	yydH	1841	2771	2876	5330	6473	4406	3887.90225255798	1.01775864581137	4.48E-07	4.37E-05
BSU21450	sunS	1808	2323	2692	7489	8798	3761	4400.71721027082	1.45897372995835	4.93E-07	4.70E-05
BSU21470	sunT	2622	4657	3898	13275	14755	6247	7441.13878226097	1.53085376558769	7.81E-07	7.13E-05
BSU40170	yydG	3503	8171	6751	14049	15952	16129	10555.1390895733	1.23695767894223	1.05E-06	9.36E-05
BSU38720	nnrA	1162	1274	1319	391	636	839	943.866659168837	-1.12370879269716	1.17E-06	0.000102338718699781
BSU33950	cggR	23532	40622	24127	13038	15873	7404	20929.8066592847	-1.37064176309467	1.88E-06	0.000155207121718197
BSU21400	yomD	91	103	88	217	265	162	151.970324860881	1.09052136077168	2.17E-06	0.000175544201782684
BSU27786	ena	668	1742	459	157	154	65	547.018932688374	-2.99694253834036	7.81E-06	0.000507471294511421
BSU17670	cotU	63	99	92	202	232	151	137.668975471662	1.1093169621192	8.66E-06	0.000523678576583695

BSU21440	bdbB	502	605	829	2119	2521	983	1238.36847816358	1.44012108468353	8.59E-06	0.000523678576583695
BSU05050	lrpA	200	387	227	579	627	554	422.268182611791	1.0264375933365	1.00E-05	0.000579999399754406
BSU35610	tuaA	2729	8112	2308	771	1154	655	2636.44554505266	-2.433751945199	2.45E-05	0.00117890745607515
BSU21420	bhlA	45	48	56	138	146	86	85.3985707811908	1.215677955038	2.90E-05	0.00127064851114843
BSU11210	argB	4288	2810	3103	1701	1481	2274	2651.6972519093	-1.00697906530462	3.22E-05	0.0013811796970992
BSU34860	hisI	7093	8416	10142	2995	3717	6604	6546.92668312427	-1.05272763594804	0.000104276949572377	0.00315107317334557
BSU35609	tuaA	255	984	232	85	116	60	289.663660738165	-2.56880704178099	0.000200872883522464	0.00504061721166603
BSU34870	hisF	5456	6773	7913	2010	2734	5322	5072.63026957704	-1.10994909304599	0.000302761371983525	0.006910367272241
BSU04370	ydaS	14055	16161	16623	4543	6823	12841	11920.9558755739	-1.06513637286362	0.0004368719718433	0.00884253599613018
BSU24980	pstC	6083	21051	4810	1732	2703	954	6248.54869930587	-2.64871642404024	0.000545871263310844	0.010009972610542
BSU15520	pyrAB	24121	25694	31372	82780	110035	26883	49246.0676588965	1.33197714519089	0.000550113851987715	0.0100448448462948
BSU15530	pyrK	7103	6881	8554	18419	26470	7771	12314.0740328762	1.11401251679196	0.00121931126246825	0.0174402154241709
BSU35540	tuaH	2290	1967	1567	6780	11364	2522	4295.09182651434	1.70783927508248	0.0037367192194734	0.0372844232603233
BSU35550	tuaG	1103	1009	878	3134	5489	1282	2090.0897776578	1.60772717241556	0.00387713094996174	0.0378108384233769
BSU24990	pstS	14810	44320	13372	5851	9206	1886	14971.0256761274	-2.18284510291882	0.00405491120446237	0.0391002786030293
BSU32490	pucC	352	112	82	70	51	94	130.712022652005	-1.46047187683969	0.00420224987105138	0.0399819383518437
BSU06460	purS	649	713	855	1819	2298	686	1153.86744973916	1.01390953169512	0.00432579532795716	0.0409756904023491
BSU35570	tuaE	1608	1278	1135	4433	6974	1708	2787.30158359318	1.58753787950331	0.00453547333014195	0.0417633391837749
BSU35560	tuaF	1062	831	679	2405	3944	1029	1620.02705888484	1.39991967503743	0.0104475137043846	0.0739574616368844
BSU35600	tuaB	4393	14634	3714	2349	3215	1593	4985.82526010814	-1.74493008129797	0.0109126948732811	0.0758936364687993
BSU32500	pucB	243	89	40	53	47	72	92.9862329901723	-1.2319322843543	0.0241982877326258	0.125751459514056
BSU15510	pyrAA	7996	9390	12374	28967	36954	9245	17203.4590496022	1.2359900857075	0.0318507078148149	0.1495310582422
BSU36510	amtB	5608	458	420	540	380	394	1369.72375239794	-2.43140779936936	0.0409260447407655	0.176317486420117
BSU24950	pstBB	1339	2295	1203	4314	7719	1321	2946.84424042925	1.3588560248527	0.05048872090285	0.19785123414989
BSU32480	pucD	1809	544	413	416	363	411	678.611917285925	-1.33576368679161	0.0521544193734676	0.201798569460369
BSU_tRNA_38	rrnW-23S	0	0	0	1	3	3	1.09958048857465	5.86054301872996	0.0525313183071809	0.202800089094565
BSU26619	yrzO	6	10	6	18	24	10	12.0973829705475	1.14262517496319	0.0537674339387466	0.205812719920751

BSU32510	pucA	411	91	81	85	71	83	141.559707506567	-1.40935260928221	0.0746119620064852	0.252492057547183
BSU24970	pstA	2210	8395	1933	1619	2535	678	2883.58711627681	-1.45517614717547	0.0775203305795056	0.258169743468259
BSU03320	nasB	3601	453	351	535	405	416	1002.76993362616	-1.82641676559524	0.0798495219327123	0.261952827685985
BSU32470	pucE	504	147	106	123	104	133	191.35435960399	-1.19180074366323	0.098119958037479	0.296860450664813
EBG00000977943	trnY-Lys	3	2	0	5	4	9	3.77240199694658	1.74728846265933	0.110653451860417	0.317176995279257
BSU03310	nasC	3269	339	340	588	353	372	917.061020611246	-1.70606058869186	0.115586835832912	0.325021698924655
BSU03330	nasA	5138	1089	1212	1395	951	1141	1880.6078943908	-1.20716864827355	0.118934872780176	0.3312249747116
BSU02690	ansZ	2448	543	364	519	503	480	834.683950984023	-1.28786214023371	0.125761711824881	0.342795824449185
BSU40780	trnY-Asp	0	0	0	2	0	1	0.510531935971536	4.72950529727493	0.19117577234406	0.437278912115331
EBG00000977897	trnY-Lys	1	3	1	1	0	0	1.02240665082351	-2.36132838193736	0.265332236885224	0.535783825164468
BSU20320	yorN	5	1	1	3	10	4	3.89053577750953	1.12898340144525	0.289875071712205	0.565064538784928
BSU_tRNA_43	rrnW-23S	1	3	0	1	6	4	2.37888116806455	1.33858267463301	0.324592971904308	0.597523999331353
EBG00000977986	rpmH	1	1	1	1	0	0	0.693474877338661	-1.6358713058057	0.478546696836613	0.719126521056928
BSU_tRNA_29	rrnW-23S	1	0	0	0	0	0	0.179623275641969	-3.32503438266173	0.486491648431615	0.723031521189743

Supplementary note 4: Pathway enrichment

BLAST-KOALA analysis

The aim of this analysis was to get all protein sequences of the genes that were differentially expressed and submit them to BLAST KOALA. This search would result in a list of KOs that can be used for iPATH visualisation.

1. The table was split into 2 tables: upregulated (Condition VS control) and downregulated genes.
2. To retrieve the sequences of the genes in each list, the tables were fed to the "Fish_GFF_info_for_fasta_v1.py" script. The script takes both the table and a GFF file (downloaded from ensemble at: ftp://ftp.ensemblgenomes.org/pub/bacteria/release-30/gff3/bacteria_0_collection/bacillus_subtilis_subsp_subtilis_str_168/Bacillus_subtilis_subsp_subtilis_str_168.GCA_000009045.1.30.gff3.gz) and compares them. Each ID in the table (column 1 - "gene_ID") appears in the gff file in the attributes section as "locus_tag". It is written into an output file called "$INPUT_FILE_BASENAME_fetch_fasta.tab".
3. The "fetch_fasta" file is then fed into the script "connection2ncbi_v2.py" script (which takes it as an argument using a "-i" switch). The script will connect to NCBI Entrez and "fish" the fasta sequence of each gene based on the GI number (of the genome), the start and end positions of the feature, and its orientation (provided in the output of step 2). Please note that NCBI requires an email address of the user: use the "-e" switch to enter your email or write it into the script to use in default cases. Outputs were named using the "-o" switch. the default is named: "./myOutput.fa".
4. The resulting output is a nucleotide fasta. feed it to "translate.py" (requires biopython) to translate it to amino acids sequence. The output will be named automatically ($INPUT_FILE_BASENAME_protSeq.fa).
5. The amino acids multi-fasta file is then submitted to BLAST KOALA (http://www.kegg.jp/blastkoala/). We used the "family_eukaryotes + genus_prokaryotes" database because if a sequence will not be found in the prokaryotes DB for any reason it might be found in the EUKs DB.
6. Out of the BLAST-KOALA results, the gene_name to KO list was downloaded (Down/UpGenes_ko_list_geneName.txt) and the KO numbers were extracted (files are named "UpGenes_ko_list.txt" or "DownGenes_ko_list.txt").

% mapping to KEGG

DownGenes: 82 KO were retrieved out of 291 genes (27.8%)
UpGenes: 142 KOs out of 206 genes (68.9%)
The lists were assembled to one tab delimited file with the hexadecimal code for the desired color to appear in iPATH for each table in this case, red (#ff0000) for downregulated genes and green (#00ff00) for the upregulated ones. The desired width of the edges is added in the last column of the table (uniform width, in this case W14)
Tables are used for iPATH visualization: http://pathways.embl.de/iPath2.cgi.
Note that the relative weights of each gene (expression levels) are not regarded. this is possible but requires another step for the analysis (the normalized weights are given in the table). it can be visualized as the width of the edge.

DAVID analysis

This analysis is examining the enrichment of functions in a list of upregulated and downregulated genes extracted from differential expression analysis performed by the bioinformatics unit of Weizmann Institute using DESeq2.

1. Gene names as they appear in the gff file were used to match a UniProt accessions (that can be identified by DAVID, in contrast to the names appearing in the files). This was done by going to http://www.uniprot.org/uploadlists/ and uploading the list of genes. Procedure is: select from "gene name" to "UniProtKB" and click "go". Than list of identifiers is downloaded (file was named "Gene_name_to_uniprot.tab").
2. Several IDs could not be found in the UniProt database (2 in the upregulated genes and 8 in the down regulated genes) - a list of "orphans" gene IDs, including gene name, product, and weather it was included in the DAVID analysis or not is provided (B.subtilis_orphan_IDs-no_uniProt_Accession.xlsx). Finally, only one of the orphans was included in the DAVID analysis from each list (from the list of the upregulated and of the downregulated genes).

    NOTE: Out of 204 upregulated genes submitted to DAVID, 201 found a hit. out of 284 downregulated genes 270 found a hit.
3. UniProt accessions were uploaded to DAVID and the CO_BP_FAT, CO_BP_ALL, KEGG_pathways, COG_ontology, and interPro_protein_domains tables were downloaded for visualization. Only GO_BR_ALL_UpGenes.txt and GO_BR_ALL_downGenes.txt were used
4. The optional parameters of the tables were first modified: all statistical values (FDR, Benferoni etc.) were included in the tables and the counts threshold was reduced from 2 to 1. The tables were saved as tab delimited text files and the headers were modified to match Shengwei's bbplot modified R script (provided - bbplot_visualization.R). The graphic is provided in the PDF file "GO_BP_FAT_enrichment.pdf". The red circles represent downpregulated and the green upregulated GO terms. the size of the circle is proportional to the number of genes assigned to the respective GO in the list and the opacity reflects the level of certainty for enrichment (the more solid the color, the more certain is the enrichment)

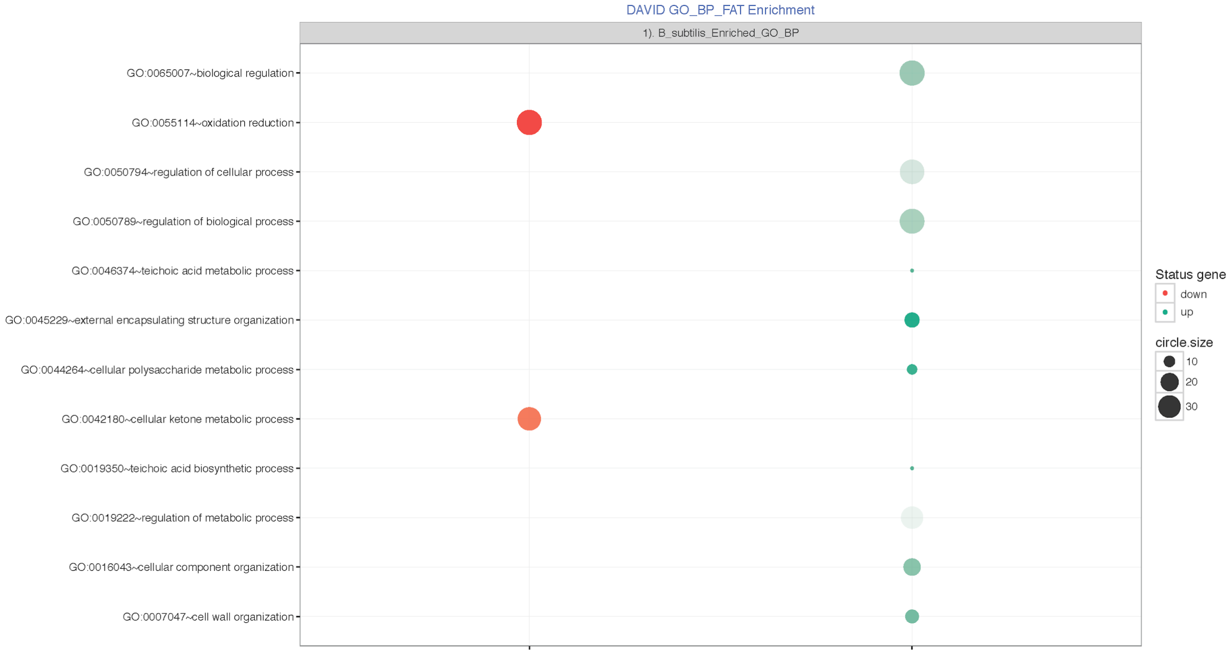

Figure S6. DAVID analysis. Circle size is proportional to number of genes assigned to the respective function, the opacity is related to the probability of enrichment of this function (the more solid the color, the more probable this function is enriched in the provided list). Red is enriched in the list of down-regulated genes, Green in the list of up-regulated. This figure is based on GO terms. It can be generated for KEGG, COG, interPro ect. GO yielded the highest amount of functions.

Table S5. KEGG ortholog list for iPATH

K01952	#00ff00	W14
K02549	#00ff00	W14
K06131	#00ff00	W14
K02337	#00ff00	W14
K00558	#00ff00	W14
K03584	#00ff00	W14
K06147	#00ff00	W14
K16705	#00ff00	W14
K00805	#00ff00	W14
K01493	#00ff00	W14
K01486	#00ff00	W14
K01447	#00ff00	W14
K07300	#00ff00	W14
K05595	#00ff00	W14
K09793	#00ff00	W14
K00963	#00ff00	W14
K03719	#00ff00	W14
K03885	#00ff00	W14
K16916	#00ff00	W14
K09888	#00ff00	W14
K16509	#00ff00	W14
K02004	#00ff00	W14
K11535	#00ff00	W14
K07727	#00ff00	W14
K03733	#00ff00	W14
K03284	#00ff00	W14
K00928	#00ff00	W14
K07025	#00ff00	W14
K03310	#00ff00	W14
K03719	#00ff00	W14
K00980	#00ff00	W14
K06380	#00ff00	W14
K07778	#00ff00	W14
K05845	#00ff00	W14
K05946	#00ff00	W14
K03090	#00ff00	W14
K06147	#00ff00	W14
K02253	#00ff00	W14
K01092	#00ff00	W14
K00963	#00ff00	W14
K03588	#00ff00	W14
K15973	#00ff00	W14
K17763	#00ff00	W14
K13256	#00ff00	W14
K01952	#00ff00	W14
K19302	#00ff00	W14
K07088	#00ff00	W14
K04757	#00ff00	W14
K01926	#00ff00	W14
K00012	#00ff00	W14
K08724	#00ff00	W14
K01732	#00ff00	W14
K19225	#00ff00	W14
K00805	#00ff00	W14
K06609	#00ff00	W14
K07217	#00ff00	W14

K03091	#00ff00	W14
K02851	#00ff00	W14
K07680	#00ff00	W14
K19224	#00ff00	W14
K04749	#00ff00	W14
K01952	#00ff00	W14
K01990	#00ff00	W14
K17828	#00ff00	W14
K18672	#00ff00	W14
K16044	#00ff00	W14
K03611	#00ff00	W14
K00525	#00ff00	W14
K07052	#00ff00	W14
K07571	#00ff00	W14
K11616	#00ff00	W14
K02823	#00ff00	W14
K03892	#00ff00	W14
K02909	#00ff00	W14
K03708	#00ff00	W14
K03317	#00ff00	W14
K19411	#00ff00	W14
K03088	#00ff00	W14
K05770	#00ff00	W14
K07038	#00ff00	W14
K04047	#00ff00	W14
K01955	#00ff00	W14
K00010	#0000ff	W14
K00016	#0000ff	W14
K00033	#0000ff	W14
K00090	#0000ff	W14
K00111	#0000ff	W14
K00123	#0000ff	W14
K00128	#0000ff	W14
K00148	#0000ff	W14
K00162	#0000ff	W14
K00167	#0000ff	W14
K00239	#0000ff	W14
K00374	#0000ff	W14
K00381	#0000ff	W14
K00382	#0000ff	W14
K00384	#0000ff	W14
K00620	#0000ff	W14
K00625	#0000ff	W14
K00626	#0000ff	W14
K00627	#0000ff	W14
K00648	#0000ff	W14
K00766	#0000ff	W14
K00782	#0000ff	W14
K00789	#0000ff	W14
K00818	#0000ff	W14
K00839	#0000ff	W14
K00865	#0000ff	W14
K00878	#0000ff	W14
K00925	#0000ff	W14
K00928	#0000ff	W14
K00930	#0000ff	W14
K00939	#0000ff	W14
K01126	#0000ff	W14
K01179	#0000ff	W14

K01265	#0000ff	W14
K01421	#0000ff	W14
K01428	#0000ff	W14
K01429	#0000ff	W14
K01439	#0000ff	W14
K01476	#0000ff	W14
K01480	#0000ff	W14
K01624	#0000ff	W14
K01661	#0000ff	W14
K01708	#0000ff	W14
K01744	#0000ff	W14
K01814	#0000ff	W14
K01872	#0000ff	W14
K01876	#0000ff	W14
K01878	#0000ff	W14
K01889	#0000ff	W14
K01890	#0000ff	W14
K01992	#0000ff	W14
K02015	#0000ff	W14
K02016	#0000ff	W14
K02037	#0000ff	W14
K02040	#0000ff	W14
K02440	#0000ff	W14
K02445	#0000ff	W14
K02446	#0000ff	W14
K02499	#0000ff	W14
K02500	#0000ff	W14
K02501	#0000ff	W14
K02518	#0000ff	W14
K02551	#0000ff	W14
K02809	#0000ff	W14
K02810	#0000ff	W14
K02863	#0000ff	W14
K02876	#0000ff	W14
K02879	#0000ff	W14
K02948	#0000ff	W14
K02952	#0000ff	W14
K02963	#0000ff	W14
K02986	#0000ff	W14
K02990	#0000ff	W14
K03040	#0000ff	W14
K03076	#0000ff	W14
K03086	#0000ff	W14
K03111	#0000ff	W14
K03169	#0000ff	W14
K03215	#0000ff	W14
K03457	#0000ff	W14
K03521	#0000ff	W14
K03535	#0000ff	W14
K03546	#0000ff	W14
K03638	#0000ff	W14
K03657	#0000ff	W14
K03753	#0000ff	W14
K03885	#0000ff	W14
K03977	#0000ff	W14
K04023	#0000ff	W14
K04091	#0000ff	W14
K04751	#0000ff	W14
K05311	#0000ff	W14

K05795	#0000ff	W14
K05845	#0000ff	W14
K05846	#0000ff	W14
K06173	#0000ff	W14
K06180	#0000ff	W14
K06416	#0000ff	W14
K06518	#0000ff	W14
K06605	#0000ff	W14
K06606	#0000ff	W14
K06610	#0000ff	W14
K06889	#0000ff	W14
K06901	#0000ff	W14
K06960	#0000ff	W14
K06966	#0000ff	W14
K07024	#0000ff	W14
K07104	#0000ff	W14
K07160	#0000ff	W14
K07217	#0000ff	W14
K07240	#0000ff	W14
K07406	#0000ff	W14
K07516	#0000ff	W14
K08166	#0000ff	W14
K08223	#0000ff	W14
K08234	#0000ff	W14
K08289	#0000ff	W14
K08680	#0000ff	W14
K09015	#0000ff	W14
K10710	#0000ff	W14
K11752	#0000ff	W14
K11755	#0000ff	W14
K11996	#0000ff	W14
K12308	#0000ff	W14
K13292	#0000ff	W14
K13612	#0000ff	W14
K13767	#0000ff	W14
K13770	#0000ff	W14
K14338	#0000ff	W14
K14665	#0000ff	W14
K15532	#0000ff	W14
K17217	#0000ff	W14
K17752	#0000ff	W14
K17758	#0000ff	W14
K17759	#0000ff	W14
K17762	#0000ff	W14
K18104	#0000ff	W14
K18928	#0000ff	W14
K18929	#0000ff	W14
K19286	#0000ff	W14

Supplementary note 5: Promoter sequences

Table S6. Tear-responsive elements

Gene	Element	Location	Sequence	Reference
skf	sigA	213824…213891	AATTTTTAGGATAATATACAAAATCCCC CTTACTTCGACAATTGCAA**TCTG**G**TATT** **AT**CGTATCGCAT	(1-3)
	spo0A			
tua	sigA	3658932...3658981	ATACCA**TTTACA**TCCAATTAACATCCGT CTGC**TAAACT**GACTGGCATAGG	(4)
	phoP	3658953...3658997	ATTCACACTTCTTAACATACCATTTACA TCCAATTAACATCCGTC	(4-6)
pst	sigA	2581674...2581724	TTCGG**TTCAAA**CCCTTTTTACATAGAAC C**TTTACT**CTATACGTGTAGGAC	(7)
	phoP	2581652...2581701	CACTGATTTACAAAACCTTAACATTCGG TTCAAACCCTTTTTACATAGAAC	(6-8)

*Bold indicates promoter regions and underlined indicates positive elements regions

1. Chen, Guangnan, et al. "Spo0A-dependent activation of an extended− 10 region promoter in Bacillus subtilis." Journal of bacteriology 188.4 (2006): 1411-1418.
2. Molle, Virginie, et al. "The Spo0A regulon of Bacillus subtilis." Molecular microbiology 50.5 (2003): 1683-1701.
3. Fujita, Masaya, José Eduardo González-Pastor, and Richard Losick. "High-and low-threshold genes in the Spo0A regulon of Bacillus subtilis." Journal of bacteriology 187.4 (2005): 1357-1368.
4. Soldo, Blazenka, et al. "Teichuronic acid operon of Bacillus subtilis 168." Molecular microbiology 31.3 (1999): 795-805.
5. Liu, Wei, and F. Marion Hulett. "Comparison of PhoP binding to the tuaA promoter with PhoP binding to other Pho-regulon promoters establishes a Bacillus subtilis Pho core binding site." Microbiology 144.5 (1998): 1443-1450.
6. Allenby, Nicholas EE, et al. "Genome-wide transcriptional analysis of the phosphate starvation stimulon of Bacillus subtilis." Journal of bacteriology 187.23 (2005): 8063-8080.
7. Qi, Ying, Yasuo Kobayashi, and F. Marion Hulett. "The pst operon of Bacillus subtilis has a phosphate-regulated promoter and is involved in phosphate transport but not in regulation of the pho regulon." Journal of bacteriology 179.8 (1997): 2534-2539.
8. Liu, Wei, Ying Qi, and F. Marion Hulett. "Sites internal to the coding regions of phoA and pstS bind PhoP and are required for full promoter activity." Molecular microbiology 28.1 (1998): 119-130.

Supplementary note 6: Silk protein sequences

>SPsegI
VAGTDGSGGALARGAAGASSVASGDGAGAASGAKTVSVAGTDGSEGAVAGGVAGASSKASGNEGSGAASGAKTV
SVAGTDGSGGALAGSAAGAISKASGDEGSGAASGAKTVSVAGTDGSEGAVAVGVAGASSKASGNEGSGAASGAK
TVSVAGTDGSGGAHAGSAAGASSKASGDEGSGAASGAKTVSVAGTDGSGGALAGGAAGASSEASGDEGSGAASG
AKAVSVAGTDGSGGALARGAAGASSVASGDGAGAASRANAGSIAGSGGPRGAVSGSSADVSSIAVGDGAALSGS
SALARSASDGAGAAVGAAQAGSVATGSGSDDSFAATNAEAGTISYGDGSLSAAQSGSIAAAKGADVNVQSGSIS
SAGNSVGAAGGSVETGQSHHCKRPVSTNSQPGGSGAEVNLPGLRIKSDAVATVGLVLHHHHHH

>SPsegII
EGSGAASGAKTVSVAGTDGSGGALAGSAAGAISKASGDEGSGAASGAKTVSVAGTDGSEGAVAGGVAGASSKAS
GNEGSGAASGAKTVSVAGTDGSGGALAGGAAGASSEASGDEGSGAASGAKTVSVAETDGSGGALAGSAAGASSK
ASGDEGSGAASGAKTVSVAGTDGSEGAVAGSAAGASSKASGNEGSGAASGAKTVSVAGTDGSGGAVAGGTAGAS
SIASGDGGSGAASRANTVSVAGSDGSGGAIAGSSADSSGIAVGDGAALSGSSALARSASDGAGAAVGAAQAGSV
ATGSGSDDSFAATNAEAGTISYGDGSLSAAQSGSIAPAKGADVNVQSGSISSAGNSVGAAGGSVETGQSHHCKK
PVSTNSQPGGSGAEVNLPGLRIKSDAVATVGLVLHHHHHH

>SPsegIII
MDFKIFLLVLLGAVVVLSAVEGRPSGDDDVRIISSTKDVTSTGKRRSNSKNNVAQSSSQKDSIRVKDSNEVIKS
ISENDPDGTRITTSKGTGVGSETLNENLVELNQNTVSDTNSVETSSDKSNSVTTYDADDRPLTVTDRKGQKDVK
SATNAKEDSSEVSENSESETNWQSESDSVEKSVSHPVCGGAESPSSGGPQGSGPQGGGSQGGPSGQGQPNGGIA
VSSAEDAAAPQSTRTSTAEGESSSFSAAADVNEGSRKGNVALAGSTAYGRSVTNENSSSGSTQSFSGAAEENVA
SPDGGSTQRNAAIAGNIAEGQVSSKEEVAEGKATSLSGTIQESDENSQRSAAIAGSSAEANVATREQTSKGKTA
AWTGNSQEKSTSPAEISETNIFSSGSYAEGSTLSREKLEQGKSTTWSEESEESRKHHHHHHH

>SPsegIV
MGSSALYLATIFLSLQVIDRSSAVAISLPRRIYPRSRPAFSLAETKILDEICSQSQCKDSCYGCFSKLSGEQQS
SESLKELKKCSSTYLGKSPYSKCQKILDNGSFNGKCDTNSTVFCDYENCLLNIELDSLVAECKLEAKADGANIF
KGTTICILTRIRCREVRFSTGYYYSPNFNYALSFNKDLKLQLFPASYLPSDGRLTCGSQDPYQPPSQAAWRNRT
CDLCHHHHHH

>SPsegV
KWKWLPKKRRLRSRKLHPQLGIPSNVFSGSPLDVLSQGPVAPGESLNAVPGSPTGFNQGSGLFPHSPLDGSSAQ
GSPSAGQSPQESPISSSNLIPLLGSSPDDLGEEYYEVEEDDGSWDYVSPDENTNSEDEHEDSSEDTNSSLPGSS
GSPPEGPSSGPNSPSGNTETGSPKNENGNSPQSRSQSSVPSLAPSSPSGSTSSPIVGKPEGDTSSSGGPSGSAS
PQVSSPGQSSQGTPGSLSGTPQDGSLSPGSPQDASSPEQSSQGAPGSSTDAPQDSSGSATKDDSPSSNGRTSKP
SSATKRPGKGIKGLTKKKKGSPKAGKWTWLRKPGKRLRKKLKPVQLKPSGIPLSQGILKPTTDVSPNVIAQGNA
AASAVAEGGKLSPNAPSPRSIAASNADANAAISSPDASLRADSAASSVASQVNNRSPLGALQGSRAEADAAAQA
VITAPNQPRQEPRVSPGSAQEKSQRGNPFDLRNSLWQDENDQFNSLSSKLNQLSKNNKHLASNINNIENIFDKL
YKFRSNTATNKHHHHHH

Supplementary note 7: Protein expression in insect and bacterial systems

Alkaline lysis of transformed *E. coli*, and subsequent acidification of the lysate (using sodium acetate) and dehydration (using ethanol) resulted in rapid assembly of disordered mats of fibers of various diameters ranging from ~1 to ~20 μm **(Figures 9-10)**.

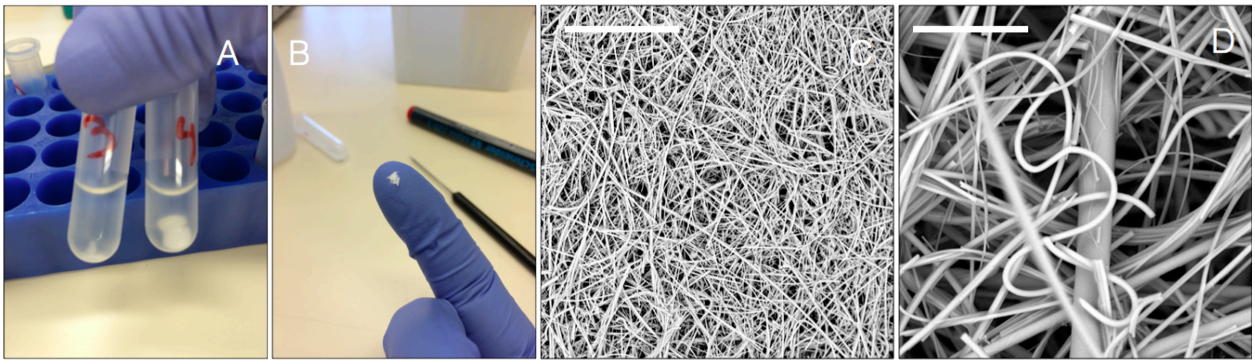

Figure S7. Assembly of silk fibers from transformed *E. coli*, following alkaline lysis, acidification, and dehydration (scale bars: C, 1000 μm; D, 100 μm).

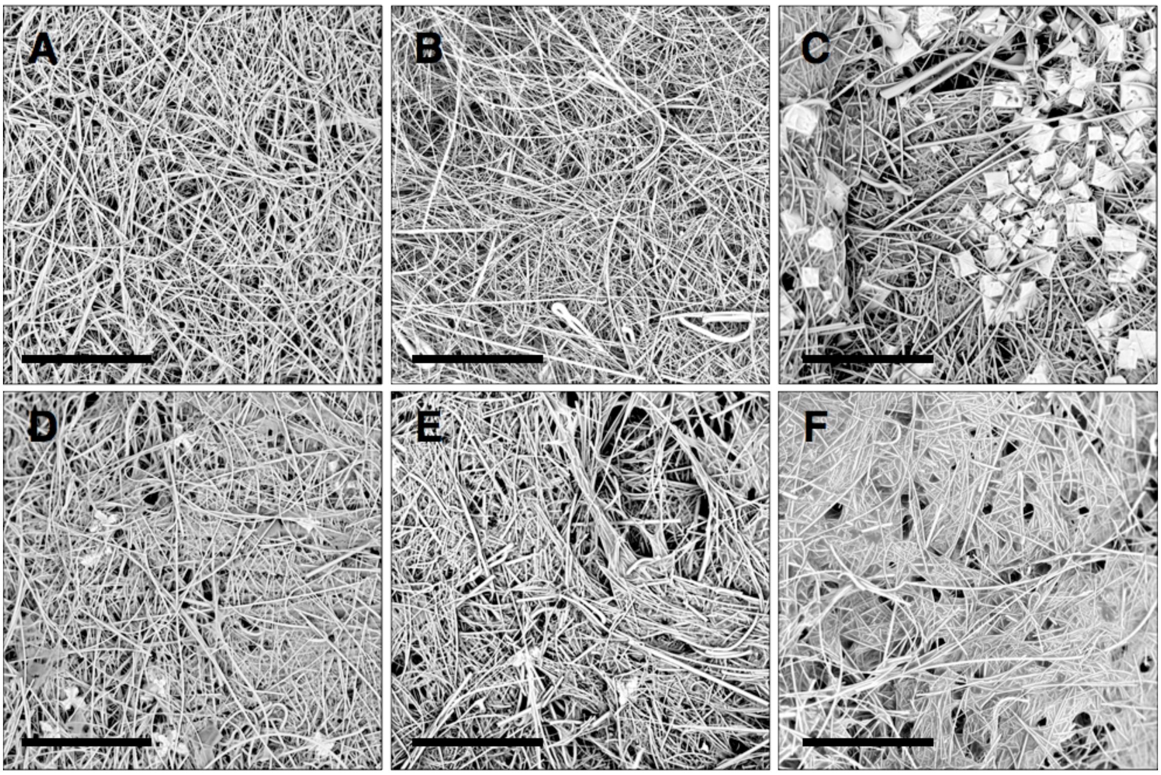

Figure S8. Silk fiber structures of single segments and a combination product. Panels A-E show structure of segments spsegI-V, respectively, while panel F shows the combination of spsegII+spsegV (all scale bars, 1000 μm; panel A is the same one used as panel C in Figure S7). Cubic objects appearing in some of the panels are salt crystals by EDS analysis.

In order to study the physical, mechanical, and chemical properties of these silk fibers, a higher-scale production was required. In addition, we decided to switch to an insect system to get fibers which are as similar to the source material as possible. Our chosen

expression system was the *Drosophila melanogaster* cell line Schneider's 2 (S2), which is a macrophage-like line derived from late stage primary cultures of embryonic cells.

S2 cells were cultured and evaluated daily for appearance and viability, to ensure <5% dead cells **(Figure S9)**. An expression vector was constructed for segment spsegII. The expression vector included three modules: BiP, a *Drosophila* secretion signal; V5 epitope for protein detection; and 6His for purification. The cells were transformed using three methods - calcium phosphate, lipofectamine, and cellfectamine, with calcium phosphate resulting in successful expression of the protein **(Figure S9)**.

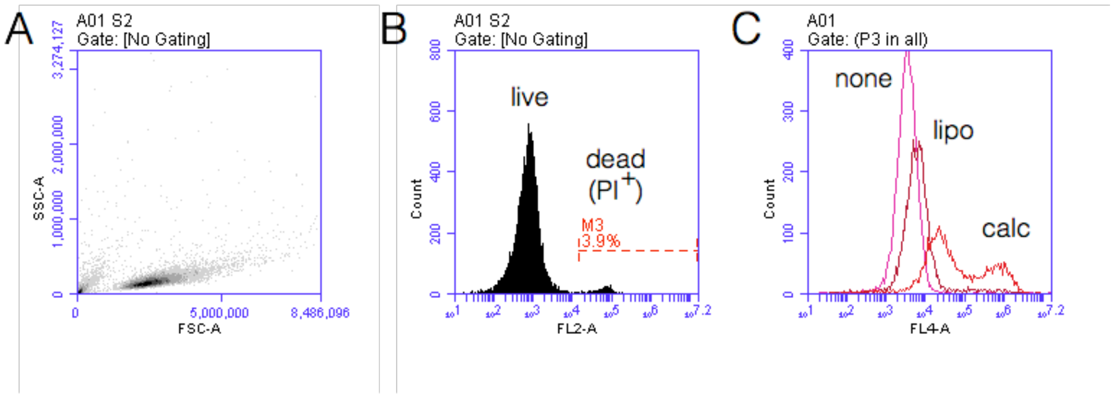

Figure S9. S2 cell culture and transformation. A, forward scatter/side scatter density plot of S2 cells. B, viability staining of S2 cells using propidium iodide (PI). C, result of transformation using either lipofectamine (lipo) or calcium phosphate (calc), compared with untreated cells (none). Cells were stained with anti-V5 antibody.

Successfully-transfected S2 cells were induced at 24, 48, and 72 hours post transfection for a period of 24 hours, after which culture supernatant and cell pellets were harvested for protein purification on a nickel column and analysis by western blot. A significant eluted fraction was detected and collected, and western blot (using weakly-reducing PAGE and anti-V5 antibody) analysis showed protein dimers in both supernatant and cell pellets from calcium phosphate transfected cells **(Figure S10)**.

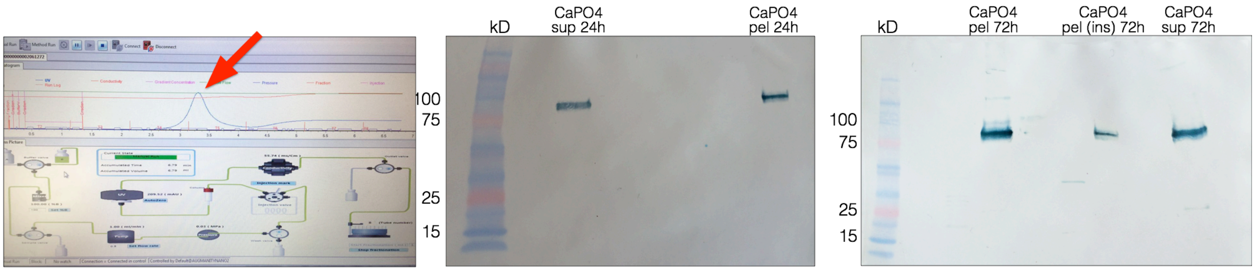

Figure S10. Protein purification and analysis. Left panel, FPLC spectrogram with red arrow pointing at silk protein elution fractions. Middle and right, western blots showing presence of silk protein in cell supernatant and pellets after 24 h and 72 h post transfection with calcium phosphate.

To produce assembled silk, purified proteins were dialyzed, concentrated, and acidified to pH ~6.0 to ~5.5. Within 1-2 min the silk protein self-assembled into fibers of ~5mm long. Fibers were analyzed for their diameter and composition using the SEM **(Figure S11)**.

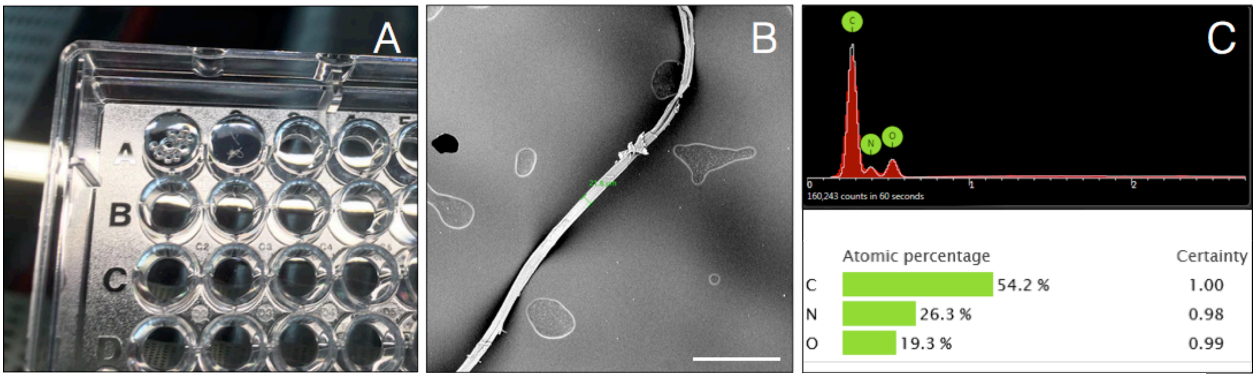

Figure S11. Silk protein expressed in S2. A, self-assembled fibers formed in well A2. B, SEM of a single fiber (scale bar: 100 μm). C, Elemental analysis of the fiber in the EDS module.

Movie S12. A Z-stack visualization of formed fiber in the fabric-biofilm hybrid.

Supplementary note 8: hypothetic crossbiotic systems

Synthetic biological products potentially address three challenges:

1. **Smartness:** biological behaviors (response, self-organization, reproduction etc.) into mechanical systems, to achieve a system that is sustainable and resilient on one hand, and flexible on the other.
2. **Precision:** biological organizations mostly occur in nature under simple and neutral conditions, compared with those in a modern studio, manufacturing facility or laboratory. Synthetic biology offers high precision ranging from the sub-nanometer scale to kilometers, without the need for sophisticated, expensive setups.
3. **Cost:** while modern machines, materials etc. often display a high level of performance (for example, a laptop), their smartness and precision are accomplished by highly complex processes, rendering them relatively costly **(Figure X)**. In contrast, synthetic biology could reduce the cost by several orders of magnitude, e.g. biofabricated computers would cost $0.5/kg instead of $500.

Biological organisms produce a remarkable array of materials with properties often far exceeding those of their synthetic counterparts including unique optical, electronic, and physicochemical properties. It is important to note, that although novel materials with information processing capabilities may be generated through physical and chemical means as well, they might be inferior in terms of sustainability and environmental compatibility than those achieved using biological, albeit synthetically assembled, parts and processes.

Table S7. Speculative crossbiotic systems constructable by synthetic biology and their applications

**Input>** **Output**	**Light**	**Chemical gradient** [O_2, water, nutrients, pH, QS, AB]	**Mechanical force**	**Temperature**	**Magnetic fields**	**Gravity**
**Fabrics**	Adaptive camouflage		Self-repairing clothing and equipment	Weather-adapting clothing		
**Adhesives/ Cements**		Water: Self-repair in sub-marine structures	Self-repair and integrity monitoring in structures			
**Foams**		QS: Biofouling-proof surfaces Water: self-insulating machines	Crack filling structures Water resistance	Self-insulating machines Weather-adapting insulation in structures		

**Hard minerals**	On-demand electronic components	Calcium/Silicates: Self-repairing structures	Calcium/ Silicates: Self-repairing structures and machines			Auto-correcting architecture (the Pisa Tower)
**Waxes**		pH: Corrosion/ humidity-proof surfaces	Crack filling structures<br>Water resistance	Self-insulating surfaces		
**Particles**	Photoactive composites and surfaces				On-demand magnetic memory devices	

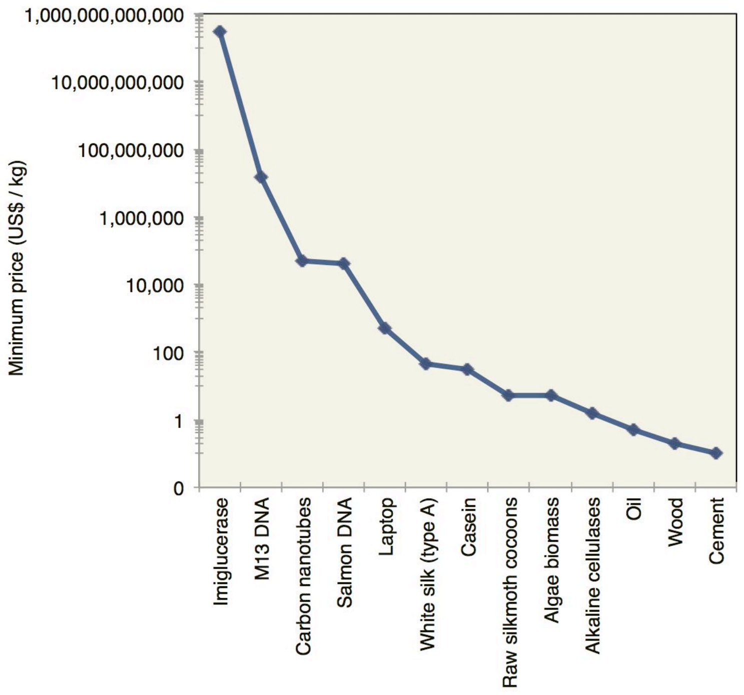

Figure S13. The cost range of a variety of usable materials across 12 orders of magnitude.
Supplementary note 9: Primers

Supplementary note 9: Primer table

Table S8. Primers used in this study

**Primer name**	**Sequence**
Oligonucleotides for promoters*	
*pst*_PhoP_F	GAG AGT **GAA TTC** CAC TGA TT
*pst*_PhoP_R	AAA AAA **CTG CAG** GTT CTA TG
*pst*_SigA_F	GAG AGT **GAA TTC** TTC GGT TC
*pst*_SigA_R	AAA AAA **CTG CAG** GTC CTA C
*skf*_Spo0A_F	GAG AGT **GAA TTC** AAT TTT TAG G
*skf*_Spo0A_R	AAA AAA **CTG CAG** ATG CGA TAC
*tua*_PhoP_F	GAG AGT **GAA TTC** ATT CAC AC
*tua*_PhoP_R	AAA AAA **CTG CAG** GAC GGA TG
*tua*_SigA_F	GAG AGT **GAA TTC** ATA CCA TTT AC
*tua*_SigA_R	AAA AAA **CTG CAG** CCT ATG CC
Oligonucleotides for confirmation of plasmid curing	
sacA FWD	CCC GAA TGG CGT GAT TTA TTG
sacA REV	CAG CCT GCC CTT TCA AAT TC

* Introduced restriction sites in the overhang shown in bold, annealing part is underlined.

Supplementary note 10: Plasmid maps

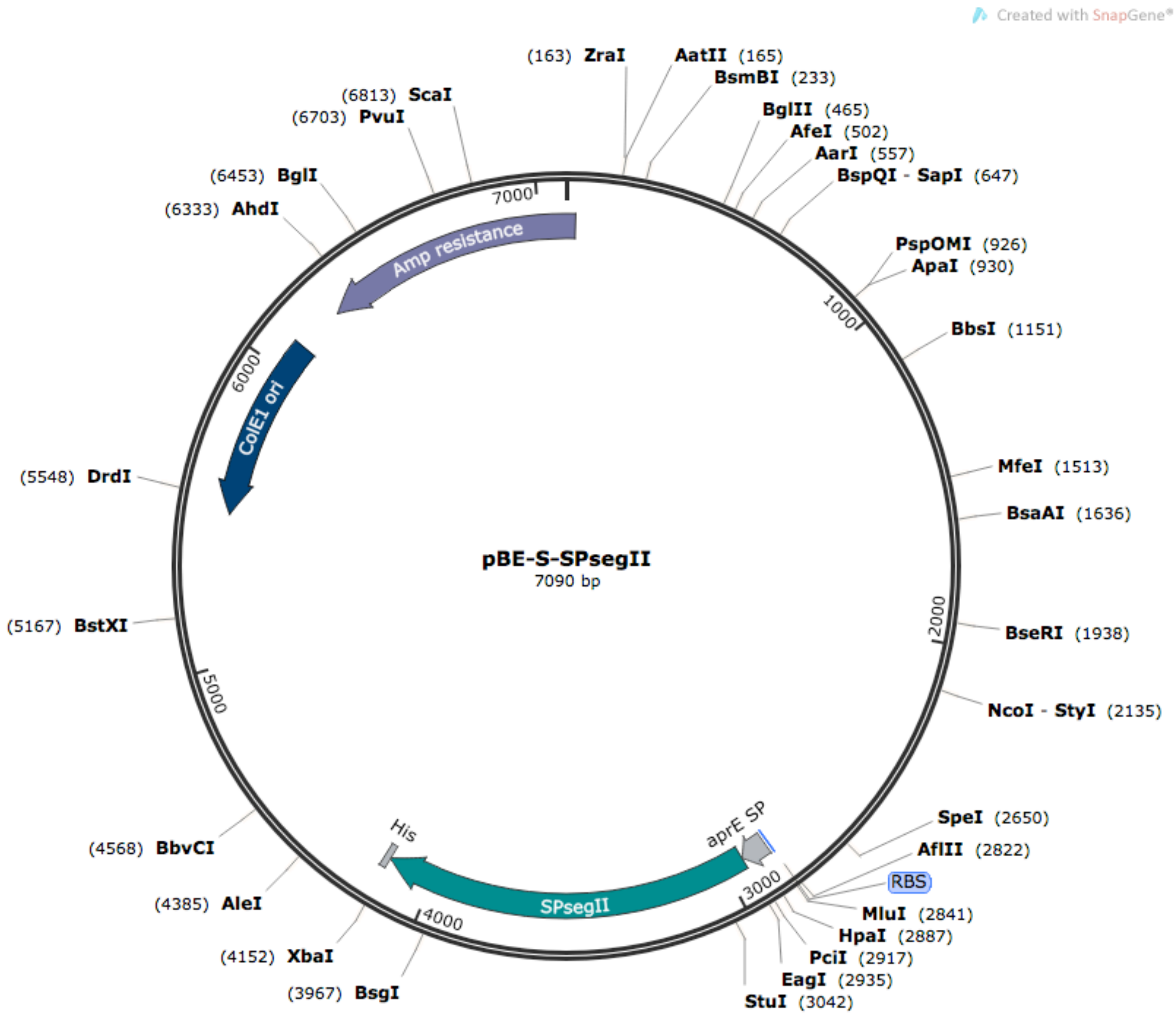

Figure S14. Plasmid map: pBE-S-SPsegII

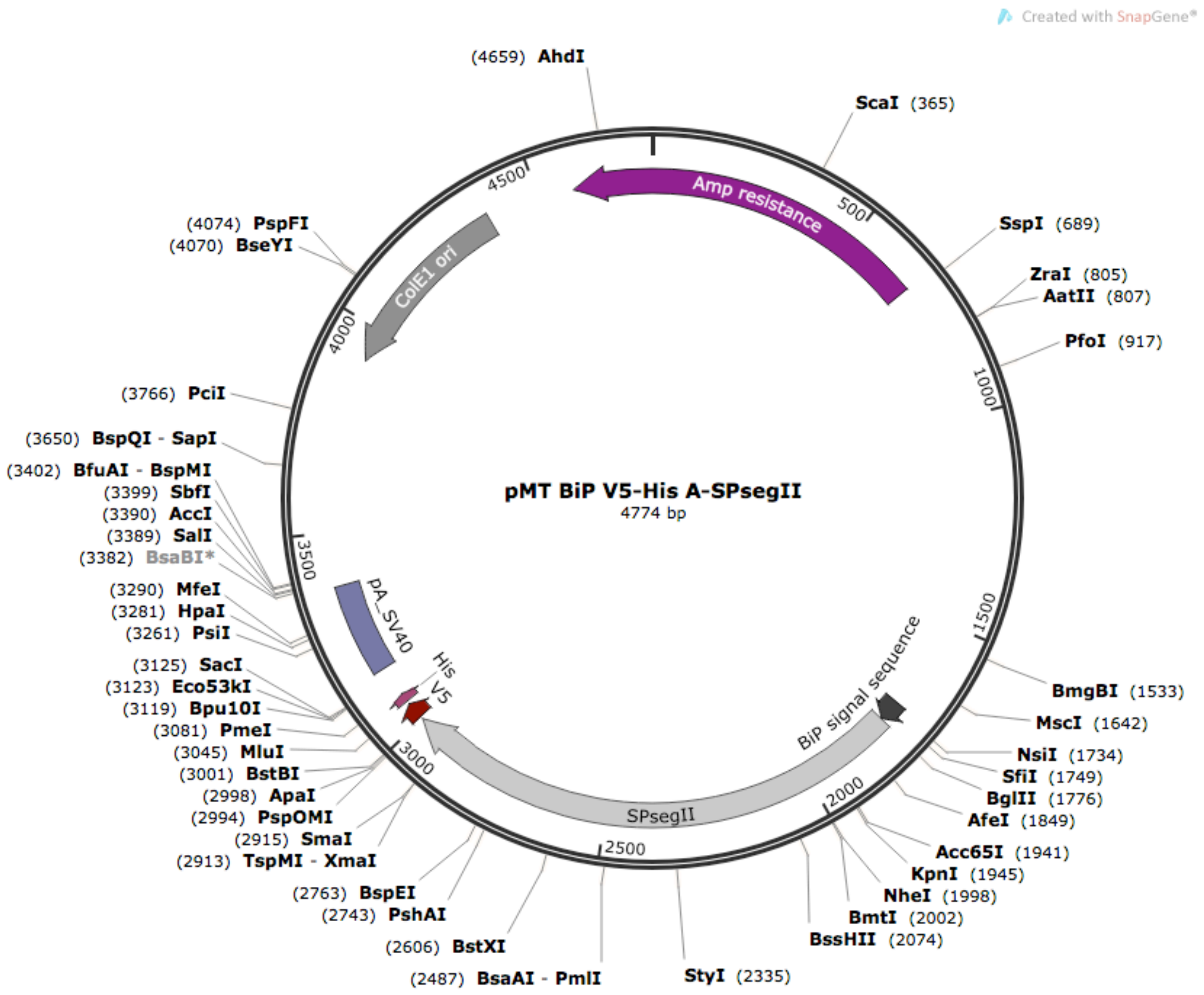

Figure S15. Plasmid map: pMT BiP V5-His A-SPsegII

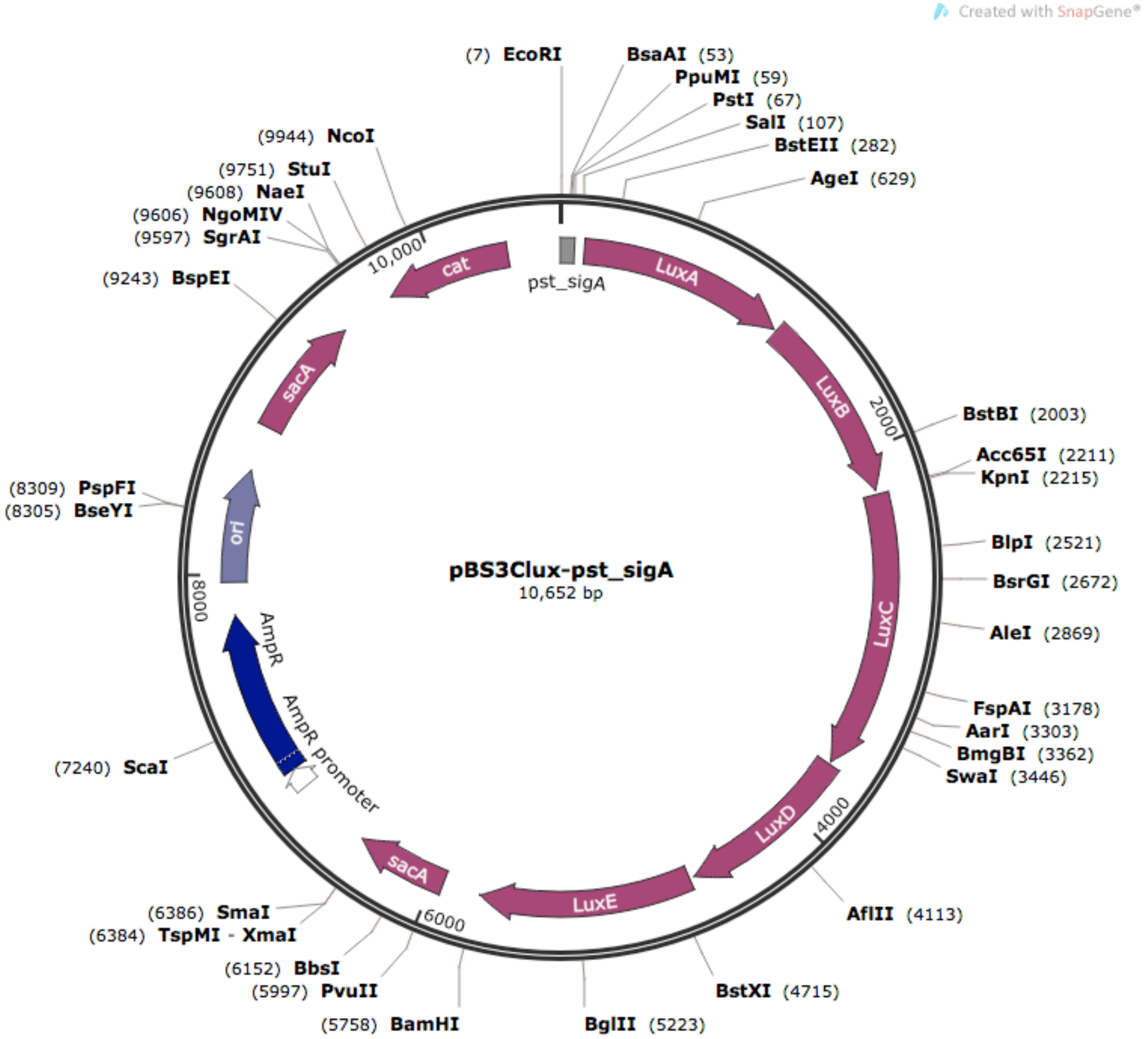

Figure S16. Plasmid map: pBS3Clux-pst_sigA. Restriction sites used to insert pst_sigA: EcoRI, PstI.

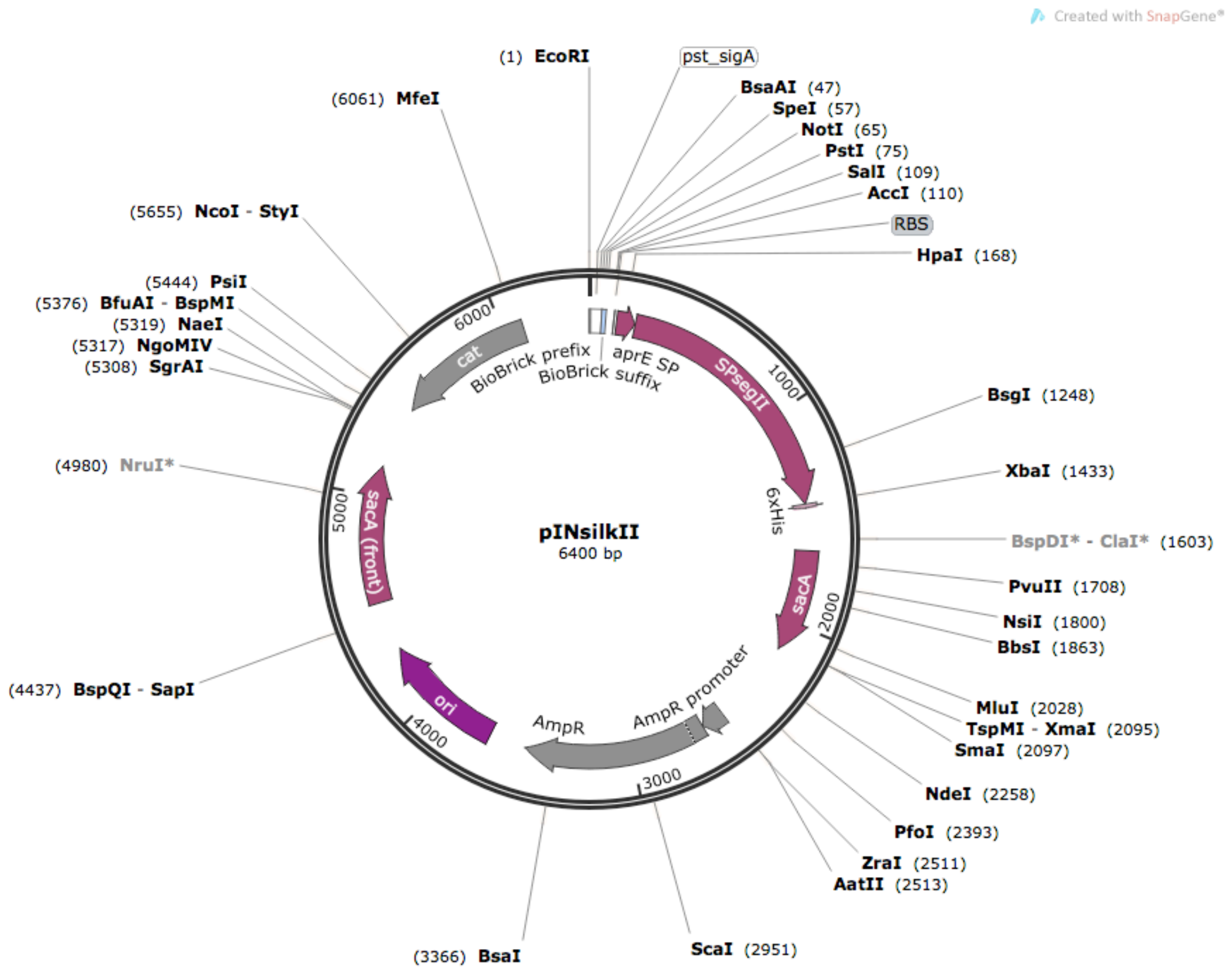

Figure S17. Plasmid map: pINsilkII. Restriction sites used: EcoRI, BglII (a new restriction site introduced in synthesis).

Supplementary note 11: Culture media and protocols

Preparation of MSgg - Biofilm inducing medium
For 500 ml of medium (x1 or for plates prepare x2, see notes below):

STEP 1 - (everything except $CaCl_2$ and $FeCl_3$):
Add to a 500ml tube the ingredients in the table below:

Table S9A. culture medium ingredients

**Component**	**Volume to add (ml) for x1**	**Volume to add (ml) for x2**	**Final conc. In x1 medium**	**Sterilizatlon by**	**Store**	**Quantity of powder for 100ml stock solution (g)**
1M K_2HPO_4	1.5375	3.075	5mM	Autoclave	RT	17.418
1M KH_2PO_4	0.9625	1.925	5mM	Autoclave	RT	13.609
0.5M MOPS pH 7*	100	200	100mM	Filter	4℃, in dark	See a detailed description below
1M $MgCl_2$ (*6H_20)	1	2	2mM	Autoclave	RT	20.330
10mM $MnCl_2$ (*4H_2O)	2.5	5	50μM	Autoclave	RT, in dark	0.19791
1mM $ZnCl_2$	0.5	1	1μM	Autoclave	RT	0.01363
10mM thiamine hydrochloride	0.1	0.2	2μM	Filter	4℃	0.33727
10 mg/ml Phe	2.5	5	50 μg/ml	Filter	4℃	1
10 mg/ml Trp	2.5	5	50 μg/ml	Filter	4℃	1
10 mg/ml Thr	2.5	5	50 μg/ml	Filter	4℃	1
50% glycerol	5	10	0.5%	Autoclave	RT	50ml
10% monosodium glutamate	25	50	0.5%	Autoclave	RT	10

*MOPS (500ml of 0.5M stock solution) prepare as follows: weight 52.33gr and add 300ml dW. Using a pH meter, titer to pH = 7.05 with NaOH 5M solution. Add more dW to reach 500ml. Filter and keep in dark at 4oC.

STEP 2 - (water and filteration):
Add dW to reach ~485ml, then filter.

STEP 3 - $CaCl_2$:

Add $CaCl_2$ as follows (to ~500ml medium):

Table S9B. culture medium ingredients (continued)

**Component**	**Volume to add (ml) for x1**	**Volume to add (ml) for x2**	**Final conc. In x1 medium**	**SterilizatIon by**	**Store**	**Quantity of powder for 100ml stock solution (g)**
0.5M $CaCl_2$	0.7	1.4	700μM	Autoclave	RT	5.549

Look for a faint opaque white cloud in the medium - a sign that everything is good...

STEP 4 - FeCl3:
Just before using the medium, add $FeCl_3$ as follows (to ~500ml medium):

Table S9C. culture medium ingredients (continued)

**Component**	**Volume to add (ml) for x1**	**Volume to add (ml) for x2**	**Final conc. In x1 medium**	**SterilizatIon by**	**Store**	**Quantity of powder for 100ml stock solution (g)**
5mM $FeCl_3$	5	10	50μM	Autoclave	RT	0.13515

If you prepare plates - add more $FeCl_3$ - about x2.5 of the amout listed above.

STEP 5 - Preparing the plates (1 Liter total):
Add 7.5gr agar to 250ml dH_2O (agar concentration of 3%) and autoclave.
After autoclave mix well with 250ml MSgg medium x2 at RT, and pour solution to the plates.
It is better to mix and pour in 50ml tube when preparing small quantities of plates - the agar tend to congeal rapidly.

Modified competence (MC) medium

Table S10. 10X MC ingredients

K_2HPO_4	10.712 g
KH_2PO_4	5.239 g
Glucose	20 g
1 M Trisodium citrate	3 ml
22 mg/ml ferric ammonium citrate	1 ml
Casein Hydrolase (from Merck!)	1 g
Potassium Glutamate / Sodium Glutamate	2 g / 1.84 g

- Mix with sDH_2O for final volume of 100 ml
- Filter
- Aliquot to 1 ml
- Store at -20°C

Procedure

1. Pick a fresh single colony from an LB plate with a sterile wooden stick to a sterile 15 ml tube containing following:
   - 900 µl dH_2O
   - 100 µl 10X MC
   - 10 µl 1M $MgSO_4$
2. Mix by vortexing
3. Incubate in a roller-drum shaker at 37 °C for 3 - 4 hr.
4. Dispense 300 µl of the sample to sterile 15 ml culture tubes.
5. Add 2 µl gDNA or 5 µl plasmid to the tube

è One tube should contain no DNA for negative control

6. Incubate in a roller-drum shaker at 37 °C for another 3 h
7. Plate sample on two different selective marker plates with sterile beads on:
   a. 250 µl
   b. 50 µl
8. Incubate plate at 37 °C overnight (when encountering problems, incubate at 30 °C)
9. Perform differential plating from a single colony with sterile wooden stick to the same selective plate as in step 7 and incubate plate at 37 °C overnight
10. If required perform differential plating from a single colony on another selective marker plate and incubate plate at 37 °C overnight
11. Pick a single colony with a wooden sterile stick into 1 ml liquid LB
12. Incubate at 23 °C overnight or at 37 °C for 2 h in a roller-drum shaker
13. Add 900 µl sample to 600 µl 50% glycerol in a freezing tube
14. Vortex well
15. Freeze the strain at -80 °C
16. Add your strain's genotype and phenotype to the IK strain collection
